# Vertically Self-Oriented, Ultrafast 1D ZnO:Li Nanorods as Scintillators for Thermal Neutron Detection

**DOI:** 10.1021/acsanm.5c03082

**Published:** 2025-10-17

**Authors:** Murat Kurudirek, Sinem V. Kurudirek, Anna Erickson, Paul J. Sellin, Mackenzie Duce, Johan Gouws, Benjamin J. Lawrie, Charles L. Melcher, Nolan E. Hertel

**Affiliations:** † School of Mathematics and Physics, 3660 University of Surrey, Guildford GU2 7XH, U.K.; ‡ Nuclear and Radiological Engineering Program, G. W. Woodruff School of Mechanical Engineering, 1372 Georgia Institute of Technology, Atlanta, Georgia 30332, United States; § Department of Electricity and Energy, Technical Sciences Vocational College, 37503 Ataturk University, Erzurum 25240, Turkey; ∥ Center for Nanophase Materials Sciences, 6146 Oak Ridge National Laboratory, Oak Ridge, Tennessee 37831, United States; ⊥ Materials Science and Technology Division, Oak Ridge National Laboratory, Oak Ridge, Tennessee 37831, United States; # Scintillation Materials Research Center, 4292 University of Tennessee, Knoxville, Tennessee 37996, United States; ¶ Department of Nuclear Engineering, University of Tennessee, Knoxville, Tennessee 37996, United States; ∇ Department of Materials Science and Engineering, University of Tennessee, Knoxville, Tennessee 37996, United States

**Keywords:** ZnO nanorods, hydrothermal synthesis, photoluminescence, ultrafast scintillators, alpha particles, thermal neutrons

## Abstract

Detection of special nuclear materials (SNMs) is of vital importance in the prevention of nuclear terrorism and to secure states’ national security. Neutron detection is a particularly useful tool to identify SNM, and neutron-sensitive scintillators have many promising properties, such as ease of use, good time resolution, and high detection efficiency. In this work, we develop highly stable, self-oriented, ultrafast 1D ZnO:Li (and codoped with Al, Ga, and In) nanorods (NRs) as thermal neutron-sensitive scintillators. Lithium-6 has high thermal neutron cross section for the (*n*, α) reaction in ZnO:Li scintillators which have a vertical nano array design greatly increasing the effective surface area and scintillation efficiency. Cost-effective low-temperature (95 °C) hydrothermal growth is used to obtain highly crystalline ZnO:Li nano scintillators by combining nuclear range data and electron transport mechanisms. Among the studies using low-temperature hydrothermal synthesis and a relatively low annealing temperature (≈350 °C) along with optimized NRs (length ≈ 5–8 μm, mean diameter ≈ 700 nm) for thermal neutron detection, this study reports the shortest scintillation decay time (≈ 470 ps) so far to the best of our knowledge. This nano array scintillator combines the advantages of a low-cost growth technique with environmentally friendly and widely available materials.

## Graphical abstract

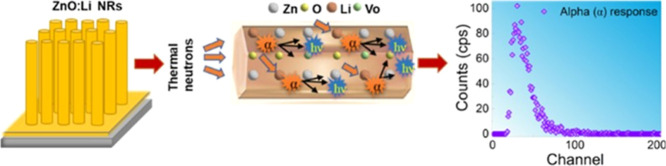

## Introduction

In the past three decades, the terrorist threats to national security have become a major concern in many countries due to their potential use of nuclear explosives, either improvised or taken from existing stockpiles, and radioactive dispersal devices. Therefore, the detection of special nuclear materials (SNM) and other illicit radioactive materials is of vital importance to the security of many countries. Beyond homeland security concerns, “detection” of SNM is also an important tool in the nonproliferation regime. Fissile materials emit low energy γ rays, which can be readily shielded by small amounts of high-Z materials. However, neutrons either emitted in high enough quantities from spontaneous fission in plutonium or generated in uranium or plutonium by active interrogation can be used to inspect objects and containers containing SNM.

Besides detection of SNM, neutron applications are progressing rapidly, requiring detectors to meet criteria such as high neutron counting rates, fast timing resolution, and low background.^1,2^ One possible application is detection of alpha particles after D–D or D–T reactions take place in a neutron generator. Luminescent alpha-particle scintillation screens are used for tagging the time and direction of individual neutrons produced by above mentioned D–D or D–T neutron generators (associated particle imaging).^3^ The current state-of-the-art neutron detectors include gas filled tubes including BF_3_ (hazardous) or He-3 (rare) gases, requiring high bias voltage to operate and are relatively large in volume. Scintillators as alternative detectors have simple technology, good timing resolution, and can be produced as higher density detector materials for high detection efficiency and good energy resolution.^4^ Recent studies clearly describe the limitations of routinely used, state-of-the-art neutron detectors as well as the advantages of new scintillators and structure designs for the enhanced detector performance.^5^

High-quality ZnO incorporated with α particle conversion layers of ^10^B or ^6^Li can be an ideal scintillator material when used in low defect density crystalline film or nanorod (NR) form. It is highly transparent in contrast to the powder form, which might be translucent due to multiple scattering effects. The potential of ZnO/LiF/ZnO sandwich structures as highly sensitive thermal neutron detectors for ^3^He replacement was previously demonstrated.^6^ It was shown that thin films of n-type doped ZnO grown by metal oxide chemical vapor deposition exhibited high sensitivity to α particle radiation, and with the placement of a 15 μm thick ^6^LiF radiator plate, enhanced detection of slow neutrons from the ZnO layer was possible.^7^

For scintillators in the form of either bulk crystals or thin films, scintillation photons can be emitted in any direction in the bulk crystal; thus, guiding these photons toward the read-out device is problematic. This will also lead to a high probability of scattering and reabsorption, resulting in poor spatial resolution. However, in a nano array design, scintillation photons will be directed to a readout device through the nanoscale structures resulting in reduced photon scattering or reabsorption. In addition, high-density arrays with a larger radiation interaction surface will increase the detection efficiency of incident radiation. Such nanoscale arrangements of ZnO scintillators were reported to have additional light guiding improvement and therefore higher spatial resolution.^8^

Many techniques are available for state-of-the-art crystal growth.^9^ However, the low-temperature hydrothermal growth discussed below has many advantages over other methods. Most of the other crystal growth methods require very high temperatures for growth (800–1900 °C) and complicated equipment and result in low light output and are more expensive. Also, powders and films may lead to assembly problems in detector installations. By contrast, low-temperature hydrothermal growth does not require complex and expensive equipment, is low cost, and produces nanomaterials with a high crystallinity at very low growth temperature (∼95 °C). In addition, the use of a wide variety of substrate materials and less hazardous chemicals as well as the potential to scale up the system for large area growth makes low temperature hydrothermal growth a good candidate for the growth of highly crystalline nanomaterials.

Doping ZnO with gallium, aluminum, indium, and lithium has been shown to adjust the shape of the optical absorption spectra. Previous studies reported in the literature have shown that such doping is possible through the addition of various nitrates to a standard ZnO NR growth solution.^10–12^ Promising advances have been made in the growth of ZnO nanoscale samples by the low-temperature hydrothermal growth method, and these samples have shown sensitivity to alpha particles.^13–16^ Following early studies made by our research group, the structural, optical, and scintillation properties of the hydrothermally grown ZnO NRs were then improved by optimizing growth parameters and n-type doping of ZnO NRs.^10,17^

Further investigations on ZnO films and nano structures demonstrated the potential of this material and geometry as a neutron detector by incorporating an ^6^Li enriched radiator, which allows thermal neutron detection via the *n*, α reaction in such systems.^1,18,19^ When doped with ^6^Li of high thermal neutron interaction probability, ZnO NRs can be highly sensitive to thermal neutrons through the following (*n*, α) reaction

1 n01+Li36→α24+H13+4.78MeV

As mentioned above, ZnO/^6^LiF composite scintillators for thermal neutron detection have been developed successfully.^18–21^ However, while the thermal neutron-α particle conversion will be significantly higher in ZnO with ^6^LiF coating compared to ^6^Li-doping in ZnO, a thick coating of LiF of more than 4 μm (where the alpha particles will be active in the layer) will lead to self-absorption of scintillation light in the coating material, which will reduce the light yield and detection efficiency significantly.

While studies on ZnO coated by Li-based compounds have been made available, the low-temperature hydrothermally grown Li-doped ZnO nano-scintillators have not been investigated as thermal neutron detectors to the best of our knowledge. In the present work, self-oriented vertically well-aligned ZnO NRs doped with Li have been successfully grown using the low-temperature hydrothermal solution technique and investigated with respect to the structural, optical, alpha, and thermal neutron associated scintillation properties.

## Experimental Section

### ZnO NR Synthesis

ZnO NRs were synthesized after deposition of a few hundreds of nanometers thick ZnO seed layer on a silica glass substrate (∼2.5 cm × 2.5 cm × 0.05 cm). After cleaning substrates ultrasonically, RF sputtering with Ar ions in a chamber was used to deposit a ZnO continuous film on the substrate. In the most common and successful hydrothermal methods developed for ZnO nanostructures growth, zinc nitrate (Zn­(NO_3_)_2_·6H_2_O) and HMTA (C_6_H_12_N_4_) were used as sources of zinc (Zn^2+^) and hydroxyl ions (OH^–^), respectively. Additives such as ammonium hydroxide (NH_4_OH) and trisodium citrate (Na_3_C_6_H_5_O_7_) were used to control the growth mechanism by coordinating to Zn^2+^ and keeping the concentration of free Zn^2+^ low. The positive (001) facet of the ZnO with hexagonal wurtzite structure has the highest surface energy, resulting in the fastest growth rate along the *c*-axis. The citrate ions present in the solution tend to adsorb on the top surface (001) and suppress the green emission which enhances the near band edge (NBE) emission even before annealing. Growth kinetics along with optimization of additive concentration in the solution growth was reported in detail in our previous work.^17^

After zinc nitrate and HMTA were added in a deionized solution with a molar ratio of 2:1, respectively, sodium citrate (1.5 mM) and ammonium hydroxide (0.8 M) were then added in the solution to control the growth. Nitrate compounds of lithium nitrate (Li­(NO_3_)), gallium­(III) nitrate hydrate (Ga­(NO_3_)_3_·*x*H_2_O), indium­(III) nitrate hydrate (In­(NO_3_)_3_·*x*H_2_O), or aluminum­(III) nitrate nonahydrate (Al­(NO_3_)_3_·9H_2_O) were then added in solution as dopants. While the molar ratios of Zn to Al, Ga, and In were kept as 0.99:0.01 along with Li as a codopant (zinc/lithium-0.9:0.1) in Li-codoped ZnO NRs, the Li concentration in LZO NRs varied between 1% and 20%. The ZnO NRs were first grown for 25 h at 95 °C to optimize the length of the NRs for α particle measurements. After optimizing growth for scintillation measurements using an alpha source, ZnO NRs were then grown for 10 h to optimize the length for thermal neutron measurements. After each growth, the substrate was rinsed with DI water and was dried at ambient air. Postgrowth annealing in a 10% H_2_ atmosphere for 45 min was conducted at 350 °C.

### Characterization

X-ray diffraction (XRD) patterns were collected on a Panalytical XPert PRO diffractometer with a step scan of 0.01 spanning a 2θ range of 5 to 80°. Cu Kα X-rays (Κα = 1.54187 Å) were used to analyze the crystal structure and phase identification. The surface morphology of the ZnO NRs was characterized using scanning electron microscopy (SEM) (HITACHI SU8230), which is equipped with a cold field emission gun for improved imaging and analytical performance. Room-temperature photoluminescence (RTPL) measurements were performed using a Renishaw spectrometer attached to a He–Cd 325 nm laser. A Cary 5000 UV/vis/NIR spectrophotometer with tungsten halogen and deuterium arc light source was used to measure UV–visible absorbance and diffuse reflectance of the ZnO NRs in the spectral range of 200 to 800 nm. A Thermolyne 21100 tube furnace was used for postgrowth annealing. X-ray photoelectron spectroscopy (XPS) (Thermo NEXSA G2 XPS with a variable spot size from 10 to 400 μm) was performed for analysis of the structure of chemical bonding using Al Kα X-rays and C 1s spectrum of carbon (BE ∼ 285 eV) as a reference. Bi ions were used to directly chemically image surfaces in ToF-SIMS (IONTOF ToF-SIMS 5) while ion sputtering of O or Cs allows depth profiles. The sample profiles correspond to 150 × 150 μm^2^ areas made with Bi^+^ ions accelerated by 50 keV and observe the masses of positive polarity ions. The depth profiling was 500 × 500 μm^2^ sputter areas (centered about the measurement region) with 2 keV Cs ions with depth resolution of 2 s sputter time. An analyzer was set to positive polarity with 256 × 256 px resolution.

Cathodoluminescence (CL) spectra from individual NRs were obtained using an FEI Quattro environmental SEM with 5 kV and 57 pA beam conditions under 250 mTorr water vapor background. Also, for comparison, a ZnO NR sample was tested under 3 kV, 76 pA, and 30 kV, 39 pA beam conditions as well. Samples were not degraded after CL measurements under the above conditions, and all the SEM and CL measurements of the samples were stable. Light collection was achieved using a Delmic Sparc cathodoluminescence module, a parabolic mirror (numerical aperture of 0.97) to collimate the light and direct it out of the SEM chamber. There, it was focused onto the slit of an Andor Kymera 193 spectrograph and measured on an Andor Newton CCD camera. To measure the fast exciton dynamics of the ZnO NRs, a pickoff mirror that directed the collimated light into a 30 μm core fiber was used. A fiber-optic Hanbury Brown-Twiss interferometer was created using a 50/50 fiber splitter where photons were detected on a pair of superconducting nanowire single photon detectors and time tagged using a Hydraharp time tagger. α particle pulse height spectra were measured using an ^241^Am alpha source (≈5.5 MeV) and a photomultiplier tube (PMT) (Hamamatsu) in a light proof setup. The live time was set to 100 s for each measurement, and the background of the measurements was tested before and after measurements. The optical response of the sample generated a cascade of electrons in an optically coupled Hamamatsu PMT with a spectral response between 160 and 650 nm. Thermal neutron measurements were conducted with two Am–Be sources (38.8 and 95.9 mCi) side by side. The sources were shielded with lead and polyethylene to minimize gamma and fast neutron flux and maximize neutron thermalization. Gamma measurements were conducted with a 144 μCi Cs-137 source with no shielding. Background measurements were taken before the source measurements. Live time was set to 1800 s for Am–Be measurements and 100 s for Cs-137 measurements. A source-to-detector distance of 1 ft was used for all measurements, and no notable detector deadtime was observed. ZnO samples were mounted to a Hamamatsu R6095 PMT with a thin layer of optical grease and secured with opaque tape to minimize light leakage and ensure a flush mount. The detector assembly was contained in a light-proof setup. A CAEN5730 digitizer was used with CoMPASS data acquisition software.

## Results and Discussion

The low-temperature hydrothermal technique used for the synthesis of vertically aligned ZnO NRs is depicted in Figure 1a. The crystal structures of ZnO NRs and typical photos of ZnO NR samples depending on the growth time are given in Figure 1b,c, respectively.

**Figure 1:**
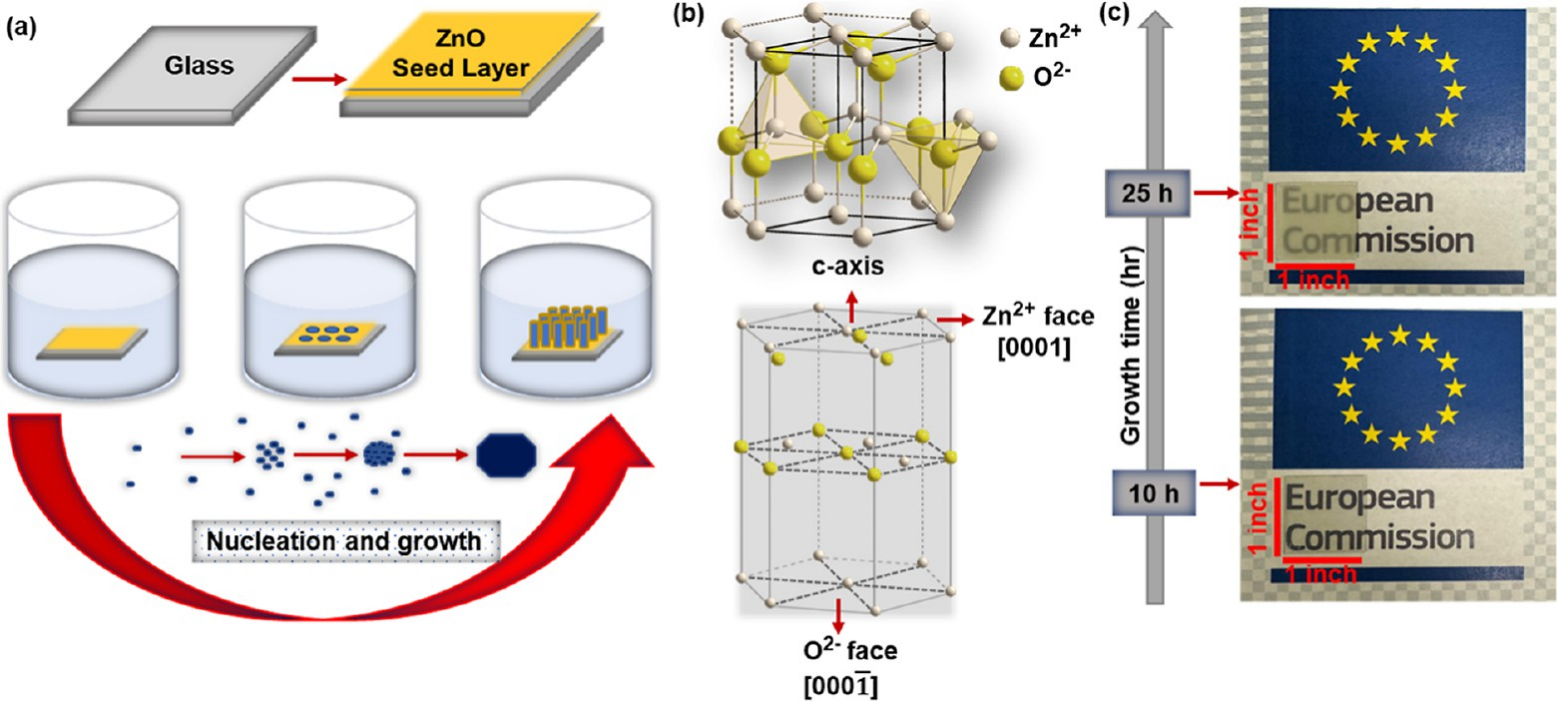
(a) Schematic illustration of ZnO NR growth. (b) Crystal structure of ZnO. (c) Photograph of ZnO NR samples depending on the growth time.

The structure of the Li-doped ZnO NRs was characterized by XRD measurements. The undoped (UZO) and Li-doped (LZO) ZnO NRs showed a crystalline wurtzite structure with a strong (002) orientation (Figure 2a). Figure 2a shows that the intensity of the (002) peak first decreases with Li doping and then becomes most prominent at ZnO:Li (10%), and subsequently, it starts decreasing with further increase in Li doping. As it is seen, the wurtzite crystallographic phase remains unaltered with the increase in Li doping although the intensity of (002) peak decreases. This might be due to the decrease in crystallinity and increase in structural defects. The enlarged XRD patterns of UZO and LZO (10%) are shown in Figure S1. The calculated values of the degree of *c*-axis alignment of ZnO NRs confirmed very high orientation through the *c*-axis (≥93%) (Figure S1c). Our results are in good agreement with the similar results reported elsewhere.^22,23^ The degree of orientation (002) is calculated using eq 1.^24^

2 $$F\left(hkl\right)=\frac{P\left(hkl\right)-P_{0}\left(hkl\right)}{1-P_{0}\left(hkl\right)}$$

*F*(*hkl*) indicates the degree of (*hkl*) orientation, *P*(*hkl*) = *I*(*hkl*)/∑*I*_0_(*hkl*), *P*_0_(*hkl*) = *I*_0_(*hkl*)/∑*I*_0_(*hkl*), where *I*(*hkl*) and ∑*I*(*hkl*) are the peak intensity and the sum of the intensities of all peaks in the ZnO NRs XRD data, respectively. *I*_0_(*hkl*) and ∑*I*_0_(*hkl*) are the peak intensity and the sum of the intensities of all XRD peaks in the reference data (JCPDS, now ICDD, 36-1451). The peak position (002) of LZO NRs showed a slight shift to lower 2θ values compared with that of UZO (Figure 2b,c). This might be due to the ionic radii difference between Li^+^ ions (0.76 Å) and Zn^2+^ ions (0.74 Å) or lattice strain, and defect-induced distortions.^25^ Since the ionic radii of Li^+^ ions (0.76 Å) and Zn^2+^ ions (0.74 Å) are comparable, Zn^2+^ ions can be substituted by Li^+^ ions in addition to the substitution of interstitial sites by Li^+^ ions, which can improve crystallinity as well.^26,27^ Crystallite size and lattice parameter were calculated using Scherrer’s equation (*D* = *K*λ/(β·cos θ)) and *c* = λ/sin θ, respectively, where *K* is constant (0.90), λ is the incident X-ray wavelength (1.54 Å), β is the FWHM, and θ is the Bragg angle.^28,29^ As the Li^+^ ions diffuse into the interstitial sites, this leads to an increase in the crystallite size and the lattice parameter is slightly increased as well (Figure 2d,e).^30^ Stress in ZnO NRs, which can be attributed to the impurities and defects in the crystal structure or growth conditions of the crystal, were calculated along with *d*-spacing values for (002) (Figure S1d).^31^ It has been seen that the LZO NRs (≥10%) with high grain sizes display significantly lower stress than UZO and LZO (<10%) NRs. Results also indicate that as lattice parameter (and *d*-spacing (002)) increases slightly, the stress values decrease confirming that the LZO10 exhibits the lowest stress among the UZO and other LZO NRs (Figure S1d). (102), (110), and (103) diffractive peaks are clearly defined for LZO NRs at higher angles, which are either in low intensity or not defined for UZO NRs (Figure S1a,b). Similar results were obtained for Li-doped ZnO elsewhere.^26^

**Figure 2:**
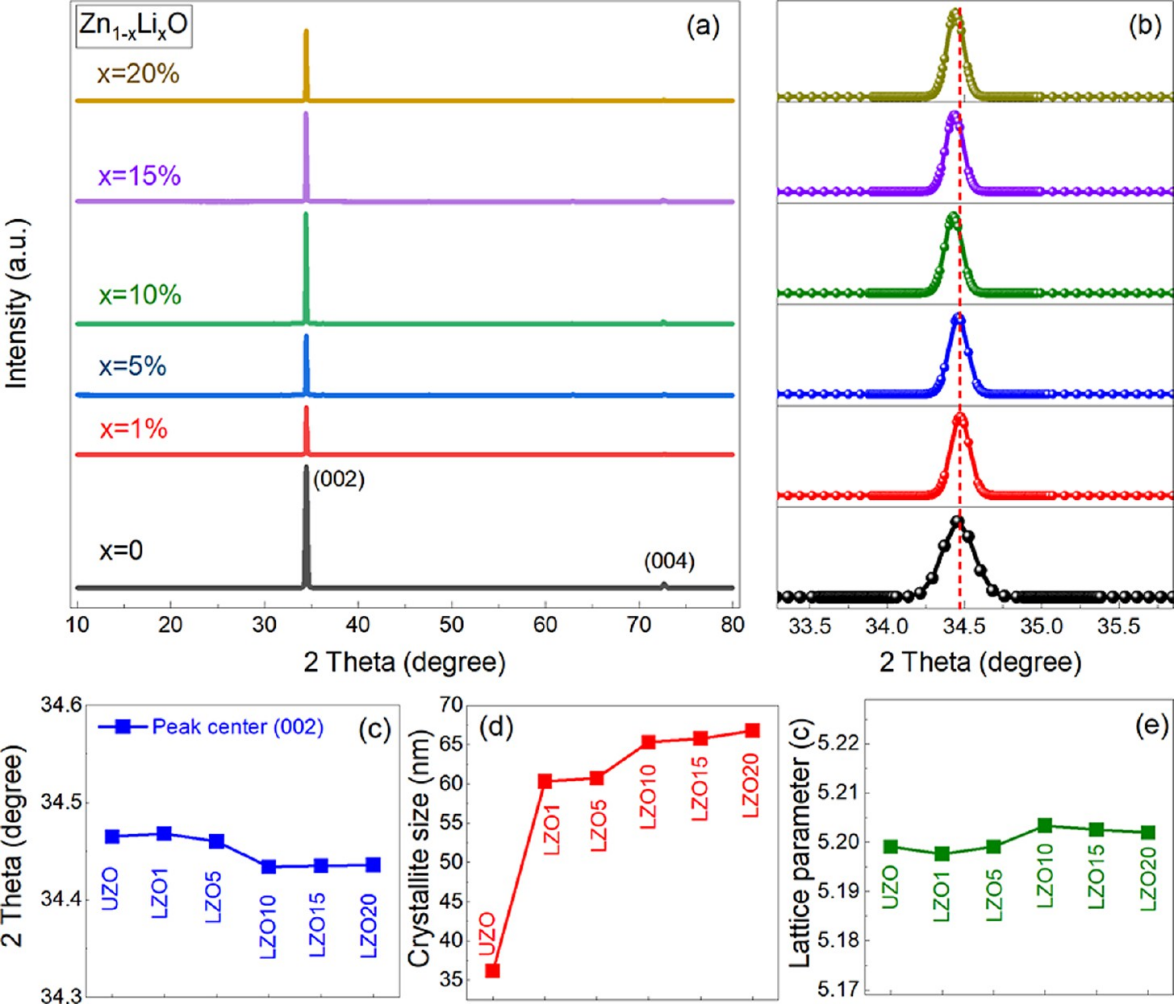
(a) XRD patterns of the UZO and LZO NRs. (b) Enlarged (002) peaks. (c) Shift in the (002) peak position. (d) Crystallite size (nm). (e) Lattice constants.

Figure 3a–h shows the tilted surface morphology and cross-sectional images of the LZO NRs of different doping concentrations (1%, 5%, and, 10%). It is clearly seen from high-magnification images that LZO nanoforests of well-aligned ZnO NRs normal to the substrate with a wurtzite hexagonal stem structure have been obtained (Figure 3i). The length of the NRs was adjusted to satisfy the scintillation measurement conditions (growth time ≈ 25 h). In scintillation measurements, discussed later in detail, an alpha source emitting ≈5.5 MeV alpha particles was first used. Therefore, the length of the ZnO NRs were first adjusted to be in the order of the range of alpha particles (≈15 μm) in the media. Due to the degradation of growth species in the solution, the growth rate slows down toward the end of the growth time, and therefore tapered NRs have been obtained (Figure 3i, inset). Scintillation measurements were then directed at growth of ZnO NRs for thermal neutron detection, which detected the recoil of the 2.1 MeV alpha particles and 2.7 MeV tritons produced from the (*n*, α) nuclear reaction. Therefore, the lengths of the NRs were adjusted to be less than 10 μm with a growth time of 10 h. Figure 4a–i shows SEM images of the Li-doped (LZO) and simultaneous incorporation of Li with gallium (LGZO), indium (LIZO), and aluminum (LAZO) ZnO NRs. All samples were doped with Li (10%) and X (1%) (X = Al, Ga, In) and exhibited vertically aligned rod shapes with little tapering toward the end of the structure. The length of the ZnO NRs was found to be in the range of ≈5 to 8 μm. The diameter of LZO NRs was found to be 0.7 ± 0.2 μm (Figure 4i). In our previous report, Al, Ga, and In-doped ZnO NRs were synthesized using the cost-effective hydrothermal method and the structural, optical, and scintillation properties were investigated in detail.^10^ Briefly, Al, In, and Ga are Group III elements which are considered as n-type donor dopants and have comparable lattice parameters to that of ZnO, which are expected to improve structural and optical properties of ZnO.^29^ They form shallow donor levels in the ZnO crystal when substituting Zn atoms and enhance optical transmission, and lead to blue-shift, thus increased band gap due to the Burstein–Moss effect.^32^ Lithium on the other hand is a Group I element and is a p-type material. However, when doped with ZnO, Li can either substitute Zn or occupy interstitial sites, which can improve crystallinity.^26^ The latter acts as an electron donor for ZnO, making it difficult to achieve p-type doping. It has been reported that the interstitial position of Li is more preferable and stable in the ZnO lattice.^33^

**Figure 3:**
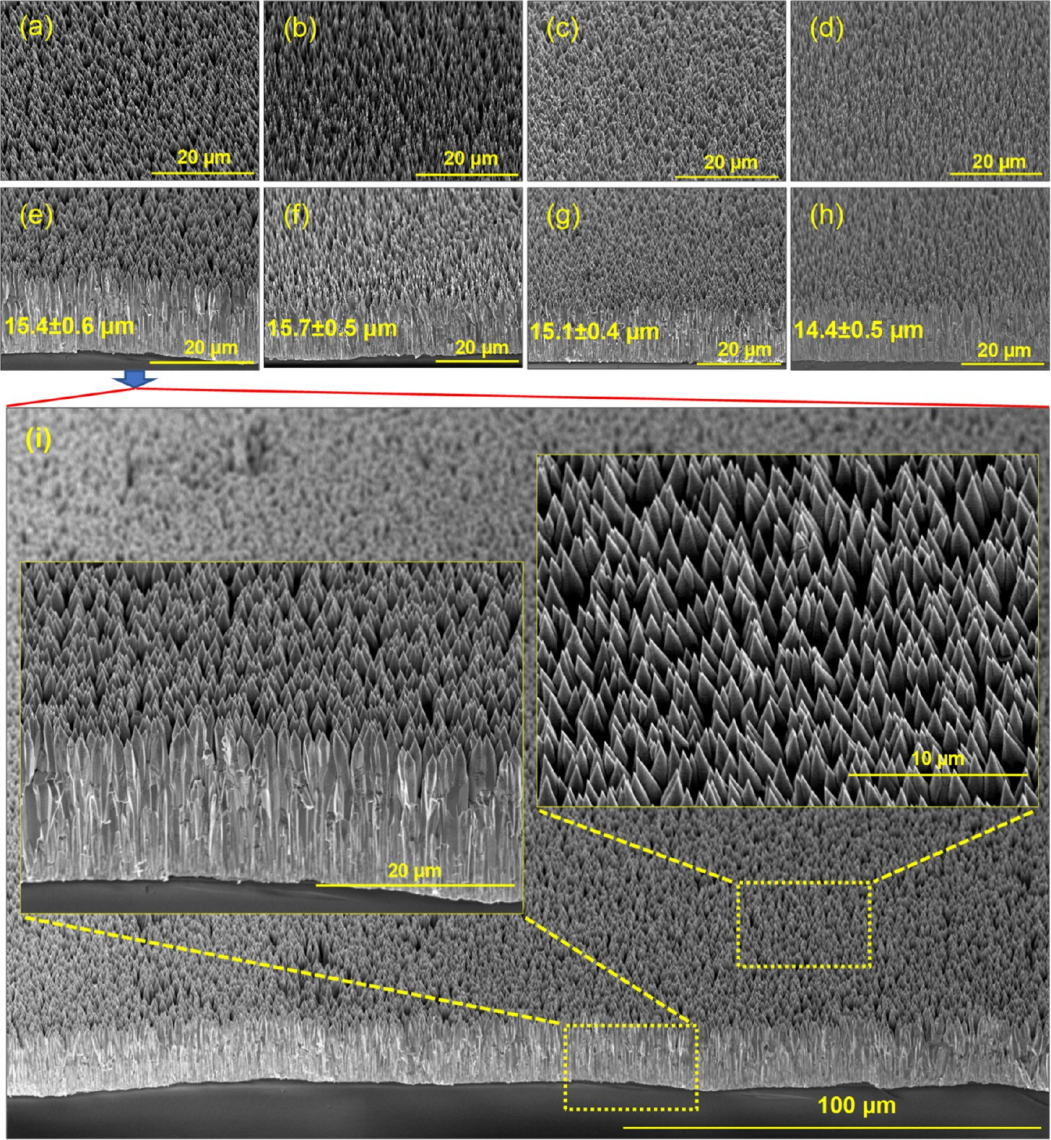
Tilted surface morphology and cross-view SEM images of different LZO nanoforests consisting of vertically aligned NRs. (a,e) LZO1. (b,f) LZO5. (c,g) LZO10. (d,h) UZO. (i) High and low magnification SEM images of LZO1.

**Figure 4:**
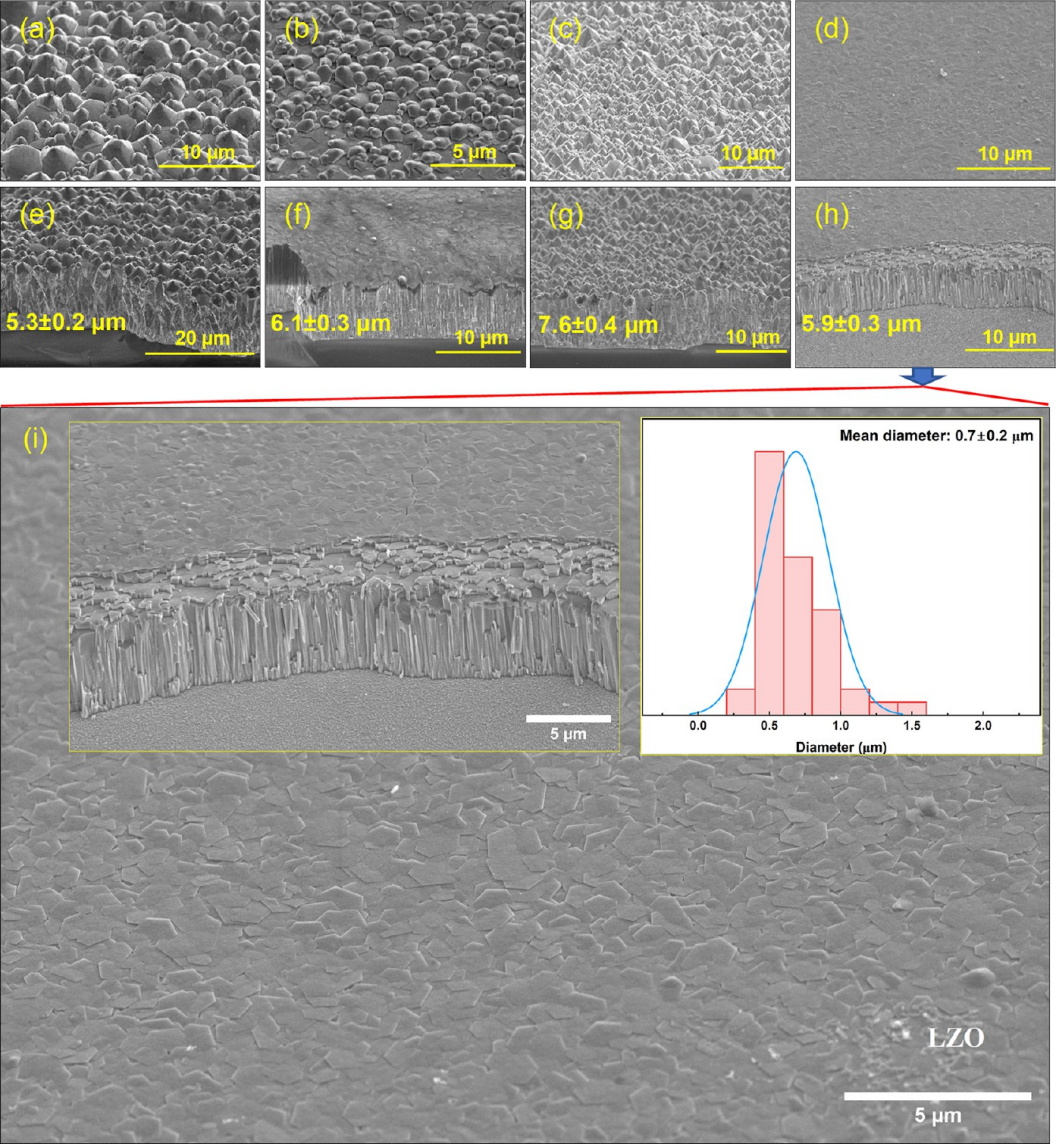
Tilted surface morphology and cross-view SEM images of doped and codoped ZnO NRs. (a,e) LIZO. (b,f) LGZO. (c,g) LAZO. (d,h) LZO. (i) Enlarged SEM images and diameter distribution of LZO (the nontapered region has been selected to calculate the diameter distribution of the NRs).

For a clear understanding of the band structure and band gap energy (EG) of the ZnO NRs, ultraviolet–visible (UV–visible) spectroscopic measurements were conducted. The optical absorbance spectra in the range of 350 to 800 nm are shown in Figure 5a,b. All ZnO NRs exhibited low absorbance, especially in the visible region. However, for doped ZnO NRs, the absorbance is seen to increase relatively in the visible region for doped ZnO NRs. This is the case for the comparison of UZO and LZO NRs with different growth time as well (Figure 5b). The absorbance of UZO with longer growth time (25 h) is higher in the visible region than that of shorter growth time (10 h), which is due to the lower light transmission for longer NRs for longer growth time.

**Figure 5:**
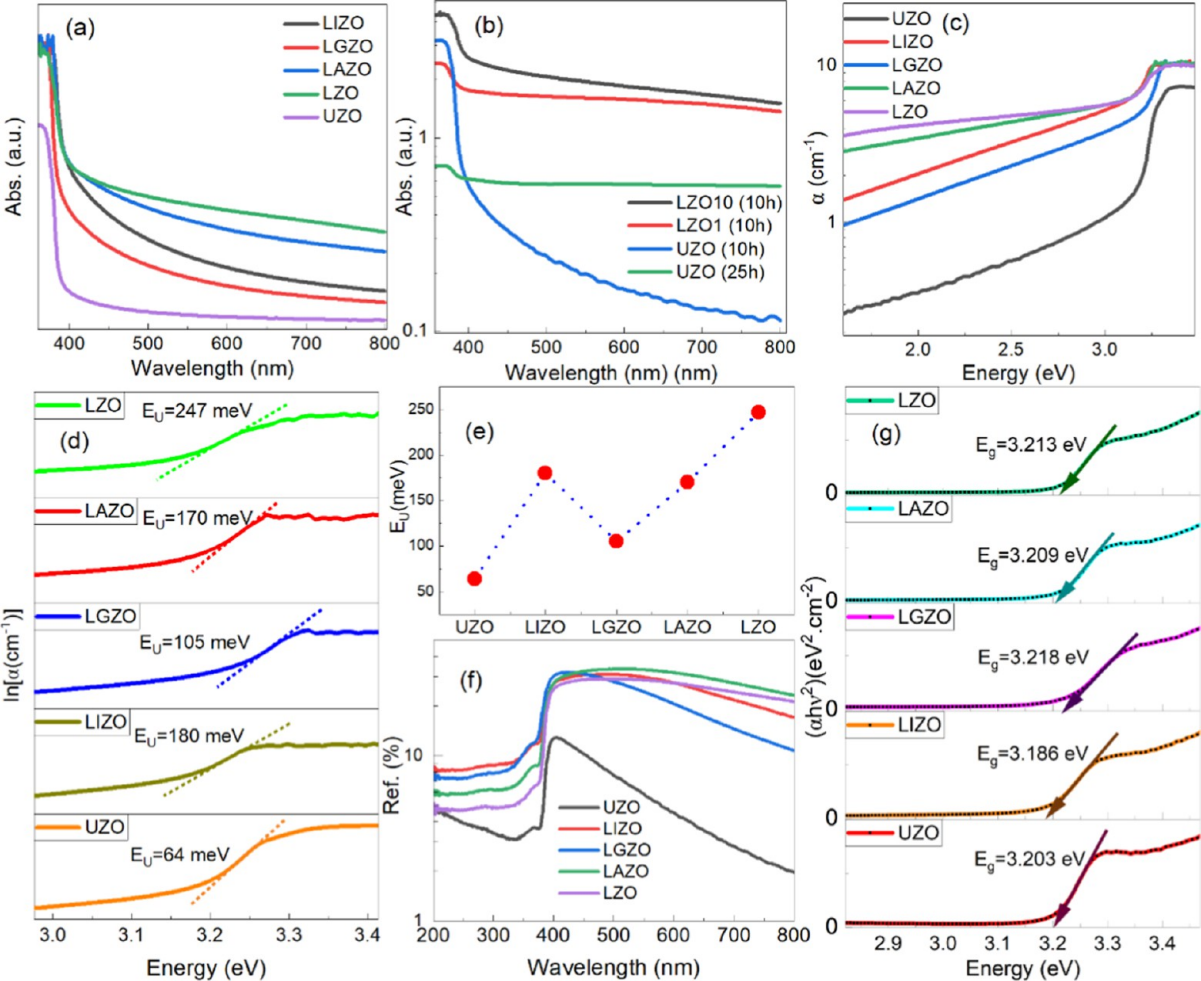
(a) Optical absorption spectra of UZO and codoped ZnO NRs. (b) Absorption spectra of LZO and UZO NRs depending on the growth time. (c) Absorption coefficient vs photon energy. (d) Variation of ln α as a function of energy (*h*ν). (e) Urbach energy (*E*_U_) for ZnO NRs. (f) DRS of ZnO NRs. (g) Tauc-plot indicating optical band gaps of ZnO NRs.

The optical absorption coefficient (α) and the variation of ln α as a function of photon energy (*h*ν) are shown in Figure 5c,d. The Urbach energy (*E*_U_) is calculated by the reciprocal of the slope in Figure 5d as follows

3 $$E_{U}={\left(\frac{d\left(\ln\alpha \right)}{d\left(hv\right)}\right)}^{-1}$$

Variation of the Urbach energy representing the width of the exponential absorption edge is also shown in Figure 5e. The Urbach energy of the UZO was found to be 64 meV, which is in good agreement with the values reported elsewhere.^29^ Due to the band tailing effect in ZnO when incorporated with dopants, the values of *E*_U_ for doped ZnO NRS have increased relatively in the range of 40% to 74% (Figure 5e). This can be ascribed to the defect states or the addition of dopant ions in the ZnO lattice.^34^ The LGZO NRs exhibited minimum *E*_U_ values among the other doped ZnO NRs, which indicates higher crystal quality and low defect states. Diffuse reflectance spectra (DRS) and the subsequent Tauc plots representing optical band gaps calculated using the Kubelka–Munk (KM) function are displayed in Figure 5f,g.^35^ All ZnO NRs showed antireflection properties as the DRS intensity was ≤30% in the visible region and much lower ≤10% in the UV-region (Figure 5f). The DRS spectra revealed that the reflectance increased with the incorporation of dopants in ZnO. The band gap energy values for the UZO, LIZO, LGZO, LAZO, and LZO were found to be 3.203 ± 0.003 eV, 3.186 ± 0.005 eV, 3.218 ± 0.002 eV, 3.209 ± 0.006 eV, and 3.213 ± 0.005 eV, respectively. In general, a blue-shift in optical band gap is observed in doped ZnO NRs, which can be due to the Burstein–Moss effect.^32^ After incorporation of dopant ions in the ZnO lattice, the Fermi level just below the conduction band of an intrinsic semiconductor shifts to higher levels due to the increase in the carrier concentration, leading to a blue-shift of the band gap value.^28,36^ For example, due to the interstitial site occupation of Li atoms in the lattice (Li → Li_*i*_^+^ + e^–^), it can produce electrons, hence the carrier concentration would increase.^37^

The results of the RTPL measurements performed for the UZO and doped ZnO NRs are plotted in Figure 6. Strong NBE emissions originating from the free excitonic recombination were observed, which confirms very high-quality hydrothermally grown ZnO NRs (Figure 6a). The NBE emission was found to increase as Li concentration increases and reached the maximum value for the LZO (10%) with 10 h growth time. As discussed later, this is suitable for thermal neutron detection due to the matching range of α particles produced from the (*n*, α) nuclear reactions. The tritons from the (*n*, α) reaction also can increase the neutron signal even though their range is much longer than the alpha particles (≈26 μm); thus, they transfer a part of their energy to the ZnO NRs increasing the scintillation efficiency. Further Li doping concentration (≥20%) leads to a reduced NBE emission, which is likely due to the lower crystallinity in high doping ratios. Therefore, LZO NRs with ≥20% doping were not considered in further measurements.

**Figure 6:**
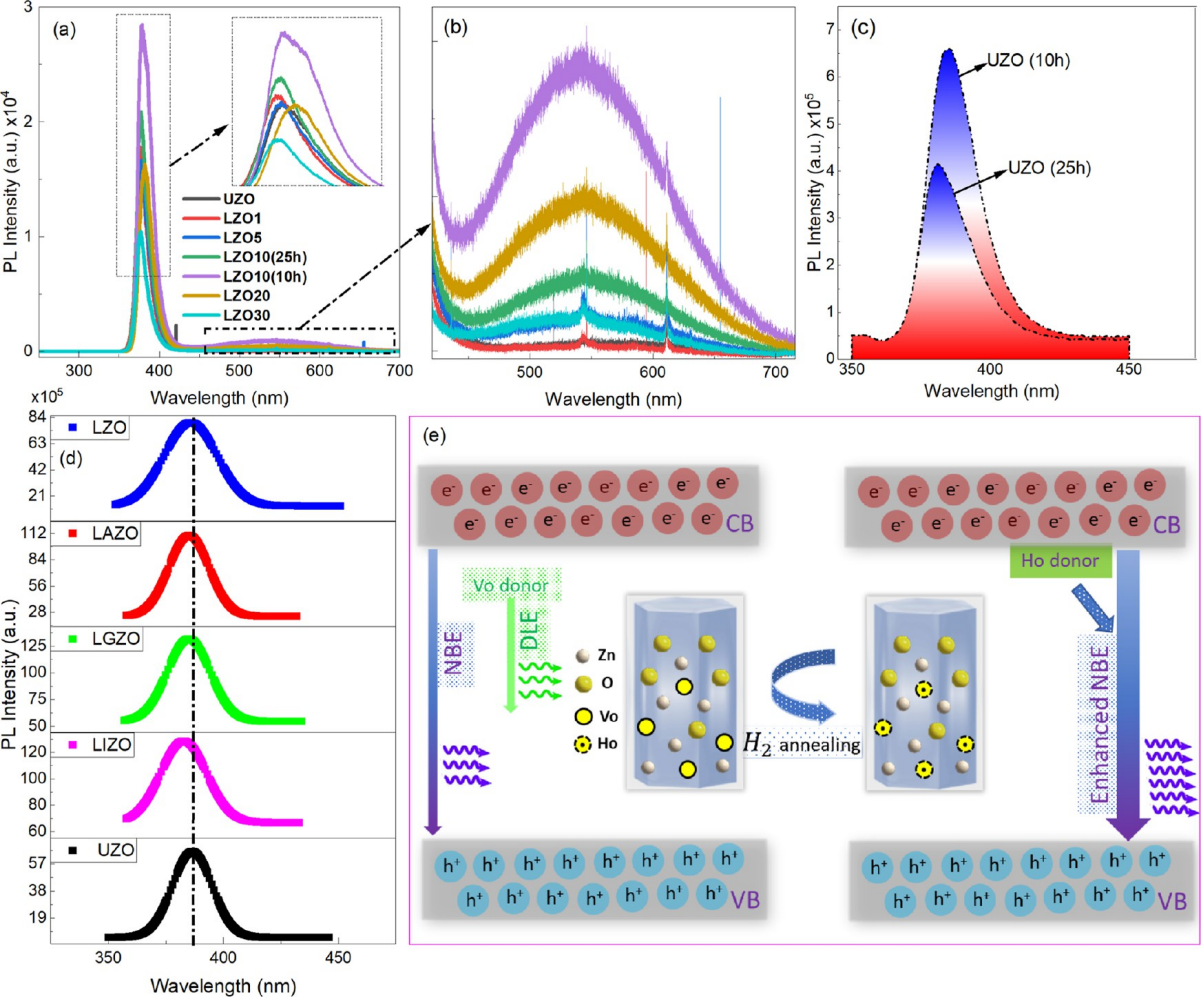
(a) The PL spectra of UZO and LZO NRs. (b) Enlarged view of green emission of UZO and LZO NRs. (c) The NBE emission spectra of UZO NRs with different growth time. (d) The NBE emission spectra of UZO and doped ZnO NRs. (e) Schematic representation of hydrogenation effect on the PL intensity in ZnO NRs.

It is observed that the longer ZnO NRs result in a relatively lower PL response (Figure 6c). This can be ascribed to the larger surface-to-volume ratio, which leads to higher surface defect states, thus quenching the NBE emission in the material.^38^ A weak green emission is also observed in ZnO NRs which increases as the Li doping increases up to 10% (Figure 6b). This deep level emission is attributed to the oxygen vacancies which originate from singly ionized surface defects.^25^ A blue-shift in the NBE PL peak position has been observed for doped ZnO NRs. As the defect intensities are significantly lower for the ZnO NRs, this slight shift is ascribed to the more electrons present at the bottom of the conduction band due to dopant ions (referred to as Burstein–Moss effect explained before) rather than the defect-driven electronic states (Figure 6d).^39^ All ZnO NRs were annealed in a partly H_2_ atmosphere defined in the Experimental section. After the hydrogenation of the ZnO NRs, the NBE emission is significantly enhanced (Figure 6a). The substitution of O vacancies (V_O_) with H creates hydrogen-donor-bound exciton levels, which strongly passivates the green emission, thus the NBE emission is significantly increased (Figure 6e).^40^ The green emission is significantly lower even before annealing with the help of a strong reducing agent, tri sodium citrate, used as an additive during solution growth of ZnO NRs (Figure S2).^10^ In our previous work, EPR measurements were performed on ZnO NRs doped with Al, Ga, and In and an EPR signal at ∼*g* = 1.96 was observed.^10^ Two EPR signals of g­(I) ∼ 2.00 and *g*(II) ∼ 1.96 are well-studied ZnO EPR signals, which were attributed to the defect states and shallow donors in the structure, respectively. A detailed investigation based on a core–shell model designates the EPR signal at *g* ∼ 2.00 as surface defects.^41,42^ Therefore, the observed EPR signal at ∼*g* = 1.96 was attributed to the shallow donors (Zn^+^ and donors Ga, In, Al). The PL intensity and alpha response (discussed later) of the UZO NRs were found nearly unchanged after an aging period of 90 days, which confirms the high emission stability of the hydrothermally grown ZnO NRs (Figure S3). Aside from being nonhygroscopic, ZnO nanostructures were found to be chemically stable even exposed to an electrochemical solution for a long time confirming its anticorrosive properties.^43^ Moreover, under harsh radiation environments including neutrons and the (*n*, α) reaction products such as alpha particles and tritons, ZnO has been found to be one of the most resistant materials to radiation damage, making it a good candidate for scintillator applications.^44,45^

The chemical structure of the LZO NRs was further investigated by using XPS. The Li 1s peaks were not well resolved as seen from Figure S4. Even for the ZnO thin film structures compared to our thick film ZnO NRs, it has been reported that the Li 1s peaks either do not appear or are barely seen in XPS measurements.^12,33,37^ A recent study reported that the Li 1s peak has not been observed until a doping concentration of up to 50%, which is beyond the scope of our research.^12^ This is because XPS is highly surface sensitive (up to a few tens of nm) and probes only the surface of the material.^12^ In such case, probing deeper into sample could work, and secondary ion mass spectrometry systems are equipped with a sputter gun to physically remove material from the sample and allow depth profiling.^37,46,47^ ToF-SIMS measurements were therefore performed to confirm the Li ions present in the LZO NRs. Results are shown in Figures S5 and S6 confirming that the Li ions are well observed and the depth profile resulted in high intensity of Li cations (Li^+^ and ^6^Li^+^).

To get more insight into the luminescence characteristics of the ZnO NRs, high spatial resolution (≈10 nm) cathodoluminescence (CL) measurements were carried out at a 5 kV acceleration voltage. Monochromatic room temperature CL images of doped ZnO NRs at NBE emission (≈390 nm) and green emission (≈550 nm) are shown in Figure 7b,c along with SEM images (Figure 7a). All ZnO NRs displayed intense NBE emission along with a negligibly small green emission as the monochromatic CL images at ≈390 nm are very similar to the SEM images (Figure 7b,c). Green emission was the lowest for Li–Ga codoped ZnO NRs (LGZO) (Figure 7c). The CL line scan mapping was performed from the bottom to the tip of an LZO NR (Figure 7d). It is clearly seen from Figure 7d (inset) that the CL emission is getting more intense toward the end of the tapered structure confirming that the NBE emission is mainly due to the top surface of the ZnO NRs.^48^ Weak green emission at the tips of the NRs indicates better crystalline structure.^49^

**Figure 7:**
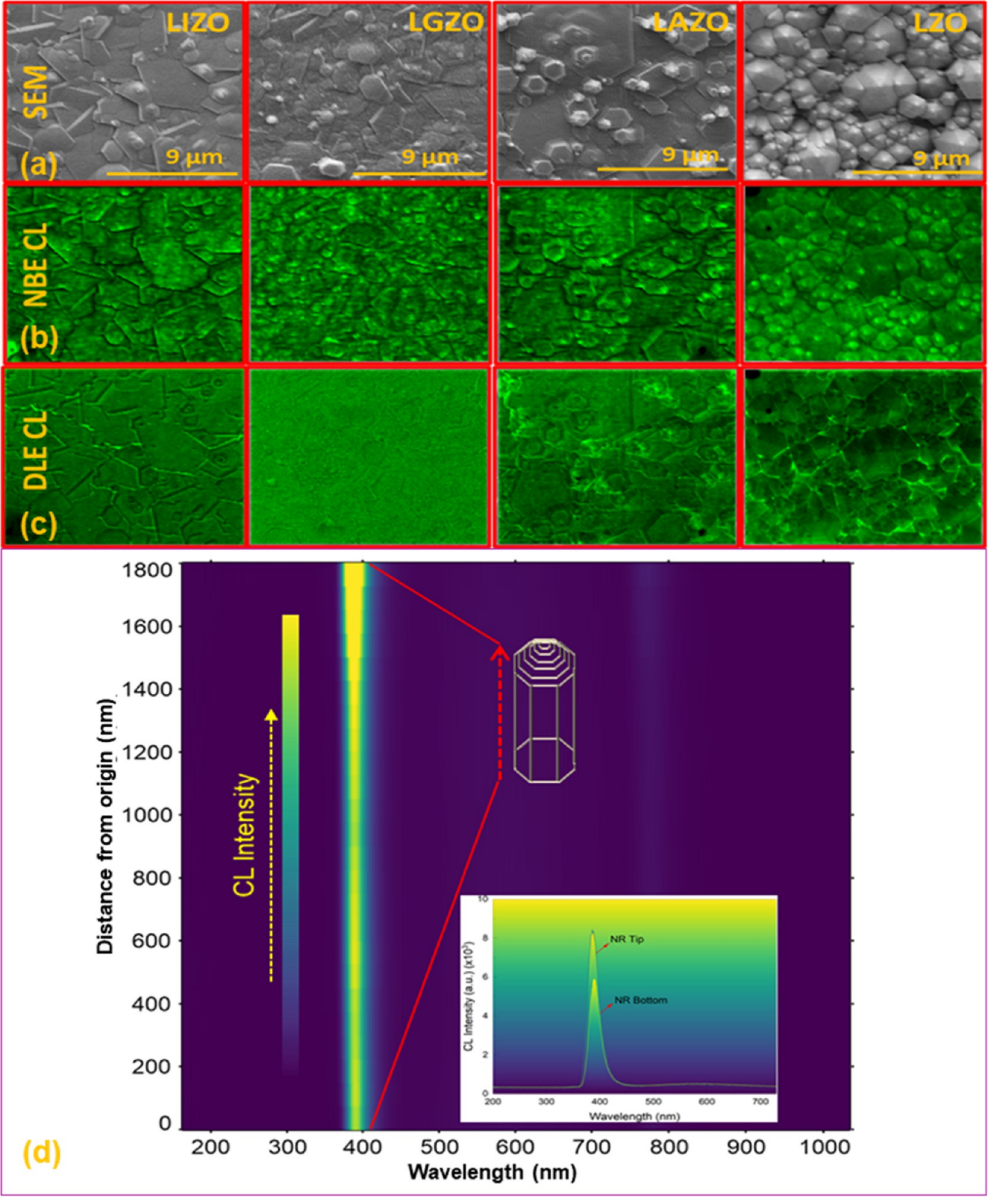
(a) Secondary electron micrograph (SEM) of the doped ZnO NRs. (b) Monochromatic CL images taken at ≈390 nm (NBE emission). (c) Monochromatic CL images taken at ≈550 nm (green emission). (d) CL scan mapping from the bottom to the tip of the NRs. The inset shows intensity of LZO NRs at the tips and bottom of the NR.

In Figure 8a, the CL spectra from the individual doped ZnO NRs are displayed. One can see that the NBE emission dominates over the whole range of wavelengths for the doped ZnO NRs. This confirms the results obtained from PL measurements. As mentioned earlier, fast timing resolution is an important parameter for nuclear detectors. The fast exciton dynamics in ZnO makes it a good candidate for scintillators used in the fast-timing application, and the diagnosis of the temporal process of pulsed radiation fields which require short decay time.^50^ To achieve a high time resolution, a ZnO crystal with short decay time is required. To probe the ultrafast dynamics of the excited states in ZnO NRs, CL photon correlation measurements were performed. In contrast to the conventional time-resolved CL measurements, the excited-state dynamics can be investigated with high spatial resolution using the CL photon bunching technique.^51,52^ The raw CL photon correlation data and the subsequent analysis of fast decay time characteristics are shown in Figure 8b,c. Temporal response of a transition radiation from a gold thin film which is used as the instrument response function (IRF ≈ 180 ps) in CL photon correlation measurements is shown in Figure S7. The best fits to the decay curves resulted in a monoexponential decay function attributed to a homogeneous excited state luminescence.^3,53^ The decay times of the doped ZnO NRs were found to be 0.91 ± 0.06, 0.47 ± 0.09, 0.70 ± 0.03, 0.62 ± 0.02, and 0.57 ± 0.02 ns for LIZO, LGZO, LAZO, LZO, and LZO1, respectively.

**Figure 8:**
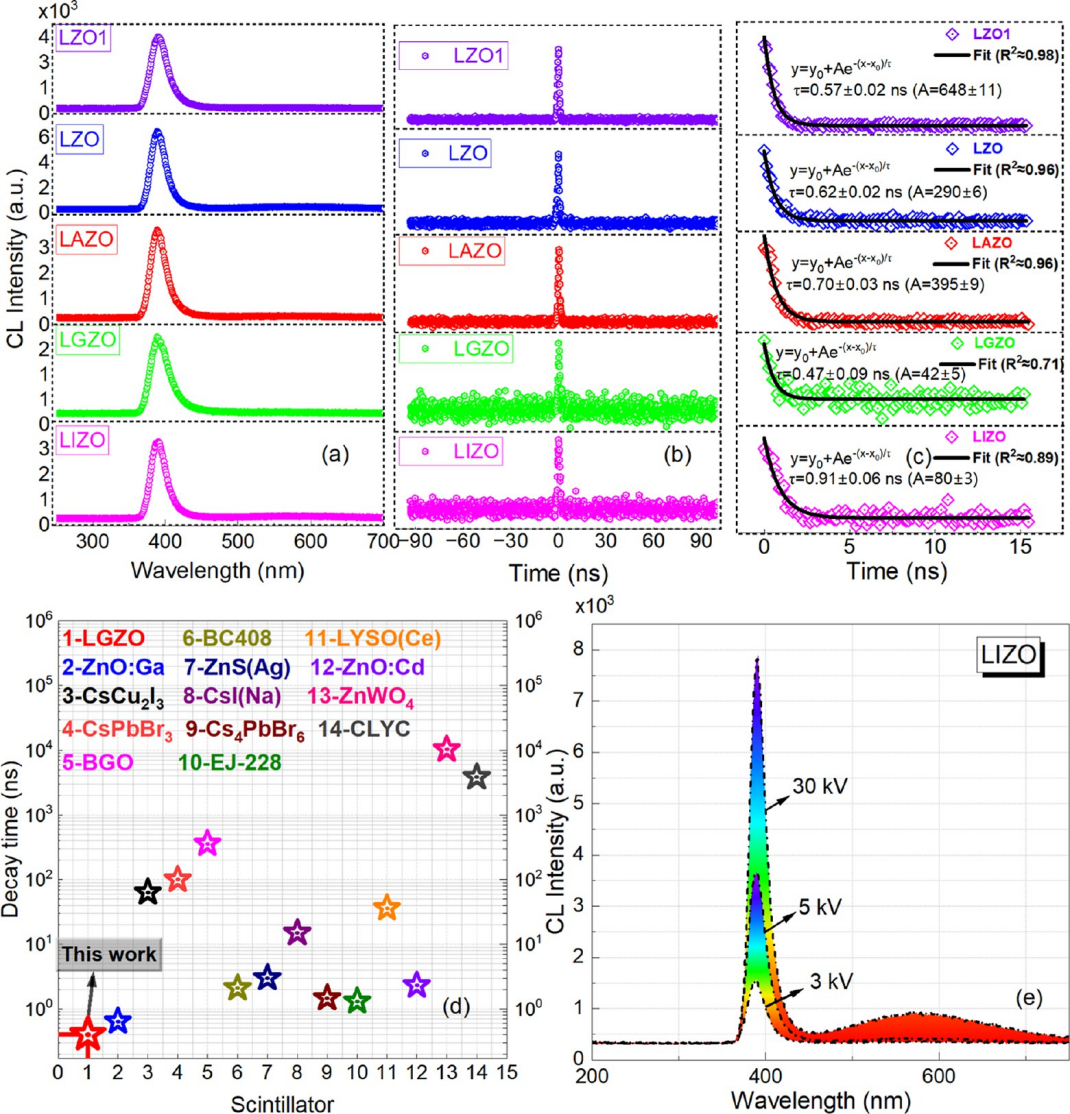
(a) CL spectra of doped ZnO NRs. (b) Raw CL data for decay time measurements. (c) Time-resolved exciton dynamics of doped ZnO NRs. (d) Decay time values reported for different type of scintillators. (e) CL spectra of ZnO NRs at various acceleration voltages of 3, 5, and 30 kV.

All the ZnO NRs display a subnanosecond ultrafast exciton dynamics behavior, which is a qualitative breakthrough compared to our previous work which reported relatively slow decay time properties for Al, Ga, and In-doped ZnO NRs.^10^ The LGZO NRs showed the fastest decay time among others (470 ps), which might be due to the increase in crystallinity and almost zero green emission as confirmed in the DLE CL image (Figure 7c). The fastest decay time observed in this work was compared with the decay times for various scintillators reported elsewhere (Figure 8d).^54–64^ There are several studies reported ultrafast scintillation dynamic characteristics in ZnO structures. ZnO and ZnO:Ga single crystals, which do not contain a NR structure, were obtained using the hydrothermal technique and exhibited mean decay time values ranging between 1 and 1.8 ns.^65,66^ Hydrothermal growth of a large area (2-in.) ZnO:Ga single crystals as α particle scintillators resulted in ∼600 ps decay time.^64^ A fastest decay time ranging between ∼60 and ∼100 ps has been reported for hydrothermally grown ZnO NRs which does not contain any α particle response measurement result to compare with the current work.^67^ Moreover, the length of the NRs is too small (<2.5 μm) to sufficiently stop alpha particles in the material and also the UV PL emission is not narrow as it contains violet and blue emissions as well. Among the studies using low-temperature hydrothermal synthesis and relatively low annealing temperature (≈350 °C) along with adjusted NR length for thermal neutron detection, this study reports the shortest scintillation decay time available to the best of our knowledge. Figure 8e shows the CL spectra of LIZO NRs as an example at various acceleration voltages of 3, 5, and 30 kV. It is seen that at a lower acceleration voltage of 3 kV, the NBE emission is by far the efficient recombination process. As the CL generation depth increases further with an acceleration voltage of 30 kV, an increase in both emissions (NBE and green) is seen, as expected (Figure 8e). However, the CL spectra at 30 kV indicate very high NBE emission when compared with the green emission and confirmed the higher crystallinity even at the core of the NRs as well. Similar results were observed which are consistent with the current study.^49,68^

As mentioned earlier, ZnO NRs with low defect density and high crystal quality are ideal scintillator material. Before thermal neutron measurements, the UZO and LZO NRs were tested for α particle responses using an Am-241 α particle source, as depicted in Figure 9a. The resulting α particle spectra collected by the detector are shown in Figure 9b. It is seen that the count rates for LZO NRs increase with increasing Li doping concentration up to 10% under the same measurement conditions, source-detector distance, etc. The count rate for LZO10 with 10 h growth time was found to be lower than that of LZO10 with 25 h growth time. However, as seen from Figure 9b, the scintillation response of LZO10 (10 h), used for thermal neutron measurements, is still comparable to the Am-241 alpha response of LZO10 (25 h), which has the highest count rate among the other samples. Based on the alpha responses, the absolute detection efficiency and minimum detectable activity (MDA) were determined. These are essential parameters in evaluating scintillators as convenient detectors. As seen from Figure 9c, the absolute efficiency is significantly increased for LZO10 NRs; thus, the MDA is significantly lower for those samples. These values were calculated using the below formulas^69^

4 $$\varepsilon =\frac{N_{s}-N_{B}}{A}$$

5 $$MDA=\frac{2.71+4.65\sqrt{N_{B}\times t}}{\varepsilon \times t}B_{q}$$

where ε is the efficiency, *N*_s_ and *N*_B_ are the net counts and background counts (cps), respectively, *A* is the source activity, and *t* is the time.

**Figure 9:**
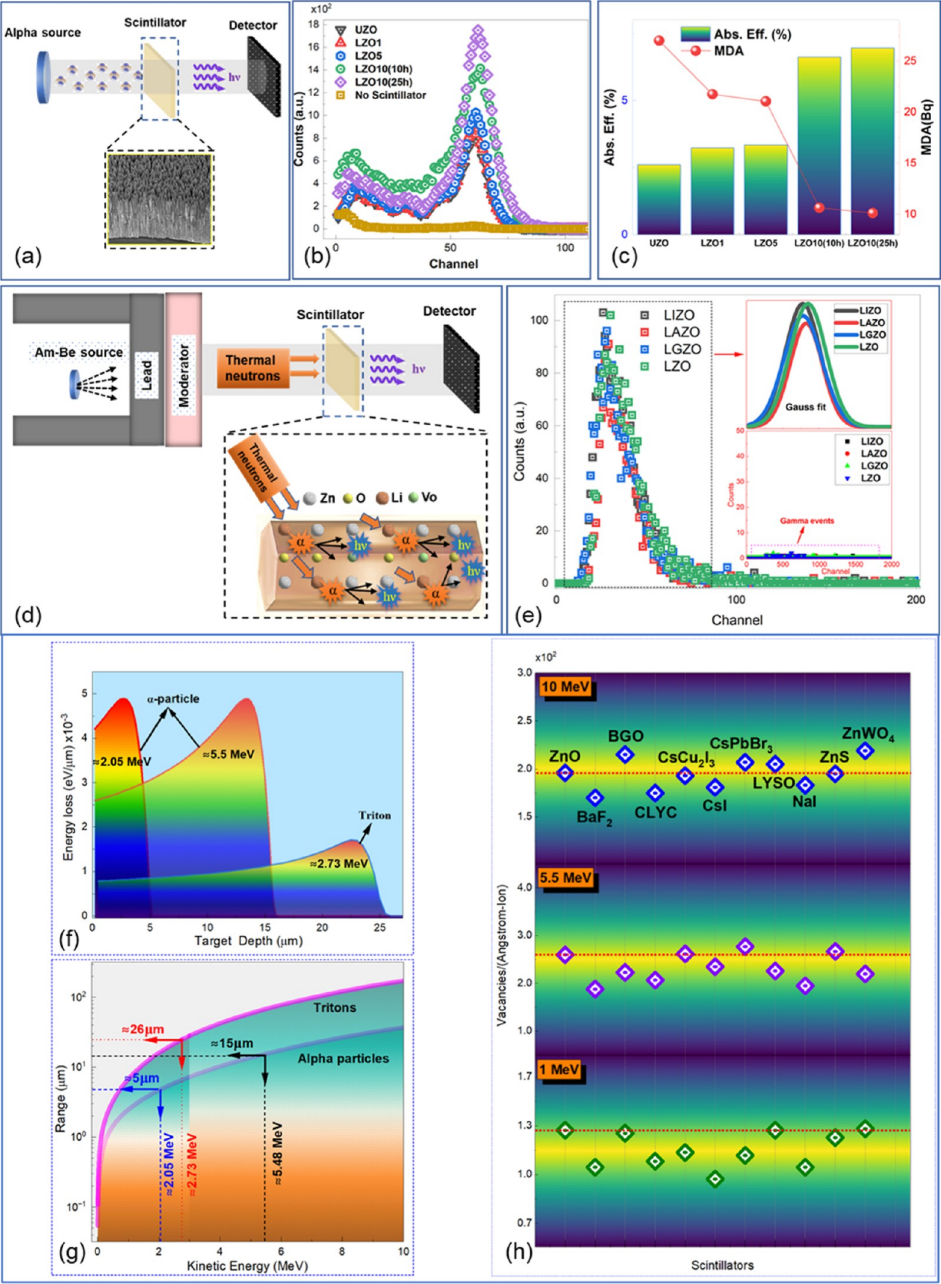
(a) Schematic diagram of α particle detection measurements. (b) α particle scintillation response of the UZO and LZO NRs. (c) Absolute efficiency (%) and MDA of ZnO NRs. (d) Thermal neutron measurements schematic diagram. (e) Scintillation spectra of alpha particles produced from thermal neutron measurements in ^6^Li incorporated doped ZnO NRs (the inset shows Gauss fit of the spectra and gamma events of ZnO NRs). (f) Variation of energy loss with target depth in ZnO. (g) Range calculations for alpha particles and tritons in ZnO. (h) Simulation of vacancies produced at various scintillators when irradiated with alpha particles of different energies.

The experimental schematic used to determine the sensitivity of the ZnO NRs to thermal neutrons is shown in Figure 9d. Figure 9e displays the α particle scintillation spectra resulting from the thermal neutron and ZnO NRs interaction. To enhance the α particle conversion efficiency, the enriched Li-6 was used instead of the Li nitrate compound in the solution. Enriched Li-doped and codoped ZnO NRs have all shown a good alpha response which is very promising for fast and efficient scintillator development for thermal neutron detectors. In the case of ^6^Li-doped ZnO NRs, the subsequent scintillation light is guided through the NRs. Due to less scattering, the signal-to-noise ratios (SNRs) are significantly higher (≥36), as seen in Figure 10a (left), and result in well-separated alpha allosteric peaks: this is not the case for ZnO/LiF composite scintillators.^18^ An MCNP model of the shielding arrangement indicates that 6% of the flux striking the detector face has energies <1 eV (Figure 10b). This corresponds to a flux of ∼47 neutrons/cm^2^/s of energy below 1 eV. Based on our measurement time of 1800 s, 84,600 neutrons (<1 eV) will strike the detector face during the acquisition period. Using the above values, the thermal neutron detection efficiency (%) has been calculated by dividing the net count rates (cps) by the thermal neutron flux and is represented in Figure 10a (right) as well. MCNP6 version 6.3.0 was used in simulation studies. This MCNP simulation represents the experimental setup used to test and characterize the ZnO NR scintillators. The setup consisted of a mixed array of lead and polyethylene bricks used to moderate a 140mCi Am–Be source. The geometry of the simulation consists of a spherical region with various material layers set inside. In order to estimate the number of thermal neutrons that reach the scintillator, the setup was recreated in MCNP and neutrons were allowed to stream from the source site to the detector site. At the detector site, the neutrons were tallied into a range of energy bins to establish the relative number of thermal neutrons present. Due to the AmBe sources used not being fully characterized, certain assumptions were made with regard to their emitted particles. All neutrons emitted in the simulation are emitted at an energy of 4.5 MeV, which is the average neutron energy of an AmBe source. The AmBe source was set up with the corrected americium activity for when the experiment was conducted, but a general correction factor was used to convert from americium activity to a neutron per second value. For ^6^Li-based ZnO NRs, the resulting α particle and tritons from (*n*, α) reaction deposit their energy locally since they have very short ranges (∼5 and ∼26 μm, respectively) in ZnO (Figure 9f,g). However, it should be noted that the range simulation assumes that the charged particles are perpendicular to the ZnO surface, which cannot be the case for the alpha particles introduced from the (*n*, α) reaction as they are not fixed in the only direction along with 1D ZnO. However, due to the NR design and total internal reflection, the scintillation light will be guided through the detector. Li ions doped in ZnO in different sites will result in an α particle range equal or less than for a solid ZnO material. Since the ZnO NRS show a highly dense distribution over the surface (Figure 4), calculated ranges can be considered as an acceptable representation of a solid and full density ZnO and gives a rough estimation of the energy loss mechanism in ZnO.

**Figure 10:**
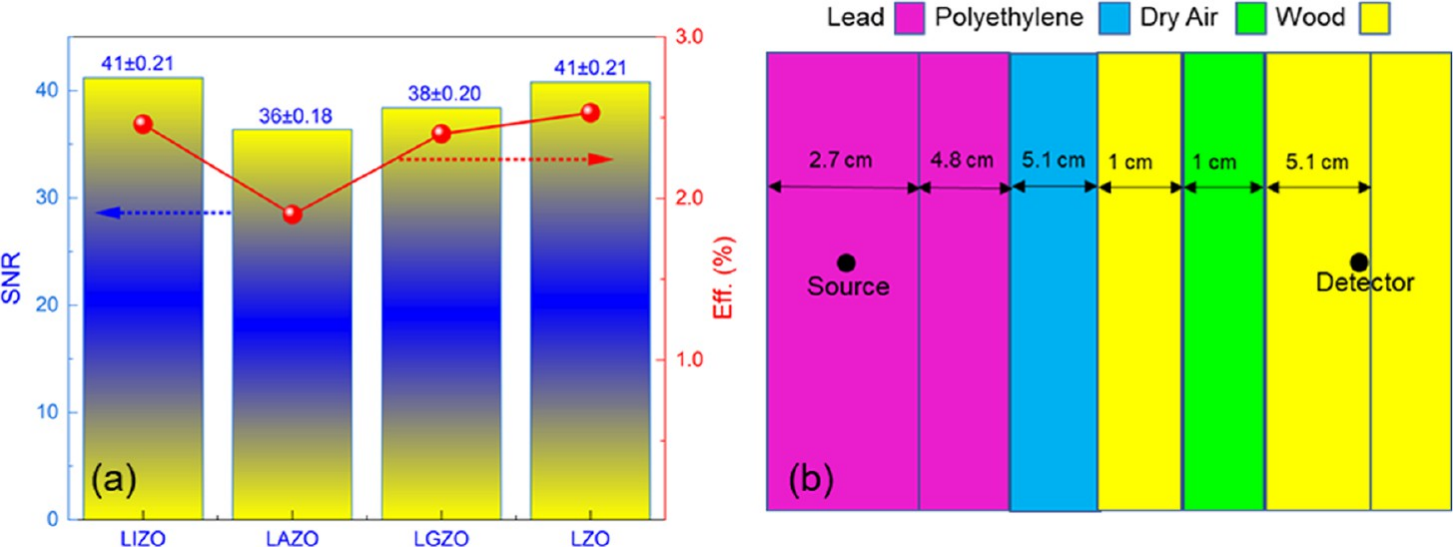
(a) SNR and thermal neutron detection efficiency (%) for scintillation spectra resulted from thermal neutron and ZnO NR interaction. (b) MCNP model of the shielding arrangements in thermal neutron measurements.

In scintillation detectors, gamma-ray and thermal neutron induced events can be readily distinguished from each other since the range of the secondary electrons from gamma-ray interactions is much greater than the grown scintillator thickness (Figure S10). In addition to the fact that the initial energy of the photo electrons from gamma events is much lower than the charged particle energies from neutron events, the electrons, whose range is much higher than the material thickness, will deposit a negligibly small fraction of their recoil energy in the ZnO NRs, resulting in a very low pulse height which is significantly less than the light output resulting from the triton and alpha energy deposition. As seen from Figure 9e (inset), the gamma events are negligible due to the thickness of the ZnO NRs (5 to 8 μm, Figure 2a) which are significantly lower than the mean free path of the gammas and ranges of the resulting electrons (Figure S8); thus, the resulting pulse height is negligibly small which is promising for neutron-gamma discrimination for the ZnO NRs.

TRIM software was used to simulate vacancy production (referred to as the hole left behind when a recoil atom is displaced) in various scintillators based on Kinchin–Pease analytic solution.^70^ The simulated vacancies produced after bombardment of alpha particles of different energies are shown in Figure 9h. Produced vacancies in different scintillators, which might lead to defects in the structure, were found to be less and close to each other since a very small part of ion energy is lost to vacancies.

## Conclusions

In this paper, the structural, optical, and thermal neutron-associated scintillation properties of Li-incorporated doped and codoped ZnO NRs are reported for the first time. The low-temperature hydrothermal solution technique was used to adjust parameters such as the growth temperature, time, solution concentration, and growth density. The ZnO NRs produced in this work have shown high crystallinity at a low growth temperature (∼95 °C). Relatively low-temperature (∼350 °C) postgrowth annealing in a partial H_2_ atmosphere did not have adverse effects on crystalline quality but enhanced the NBE emission significantly. Vertically oriented nanoarray design with optimized size (length ≈ 5–8 μm, mean diameter ≈ 700 nm) resulted in proper light absorption, guiding to the detector. Successfully incorporating Li in ZnO NRs to initiate (*n*, α) nuclear reactions has led to promising advances by demonstrating sensitivity to thermal neutrons. Our findings highlight that the further improvement in Li incorporation in ZnO NRs can be a promising approach to develop efficient detectors for thermal neutrons.

## Supplementary Material



## Data Availability

The data that support the findings of this study are available from the corresponding author upon reasonable request.

## Notes

XRD patterns of LZO and UZO, PL intensity of ZnO NRs before annealing, PL and alpha response of UZO NRs, XPS spectra of LZO NRs, ToF-SIMS results for LZO NRs, ToF-SIMS depth profile for LZO NRs, IRF CL spectrum, and range of gammas, electrons, and alpha particles in ZnO (PDF)

## References

[1] Sykora G. J., Mann S. E., Mauri G., Schooneveld E. M., Rhodes N. J. (2024). Review of thermal neutron scintillators: Evaluation
metrics and future prospects for demanding applications. Opt. Mater.: X.

[2] Mann S. E., Schooneveld E. M., Rhodes N. J., Mauri G., Liu D., Jeff Sykora G. (2024). Nanoparticle
ZnS:Ag/6LiF—a new high count rate
neutron scintillator with pulse shape discrimination. J. Phys. D: Appl. Phys..

[3] Bourret-Courchesne E. D., Derenzo S. E., Weber M. J. (2009). Development of ZnO:Ga as an ultra-fast
scintillator. Nucl. Instrum. Methods Phys. Res.,
Sect. A.

[4] Wolfertz A., Gustschin A., Schulz M., Long A. M., Khaplanov A., Hirsh T. Y., Nomerotski A., Morgano M., Tremsin A., Mauri G., Sykora G. J., Losko A. (2024). LumaCam: a novel class
of position-sensitive event mode particle detectors using scintillator
screens. Sci. Rep..

[5] Marshall M. S. J., More M. J., Bhandari H. B., Riedel R. A., Waterman S., Crespi J., Nickerson P., Miller S., Nagarkar V. V. (2017). Novel Neutron
Detector Material: Microcolumnar LixNa1–xI:Eu. IEEE Trans. Nucl. Sci..

[6] Connors, B. J. ; Summers, C. J. ; Klein, B. ; Burgett, E. A. ; Hertel, N. E. ZnO thermal neutron scintillators designed for high sensitivity and gamma-ray discrimination. 2011 IEEE Nuclear Science Symposium Conference Record, 2011.

[7] Burgett, E. A. ; Hertel, N. E. ; Nause, J. E. ; Ferguson, I. Thin Film Doped ZnO Neutron Detectors. US Patent 20,110,266,448 A1, 2011.

[8] Izaki M., Kobayashi M., Shinagawa T., Koyama T., Uesugi K., Takeuchi A. (2017). Electrochemically
Grown ZnO Vertical Nanowire Scintillator
with Light-Guiding Effect. Phys. Status Solidi
A.

[9] Nishinaga T. (2015). Progress in
art and science of crystal growth and its impacts on modern society. Jpn. J. Appl. Phys..

[10] Kurudirek M., Kurudirek S. V., Erickson A., Hertel N., Lawrie B. J., Tratsiak Y., Klein B., Melcher C. L., Summers C. J., Sellin P. J. (2025). Synergistic effects of Al, Ga, and In doping on ZnO
nanorod arrays grown via citrate-assisted hydrothermal technique for
highly efficient and fast scintillator screens. Discover Nano.

[11] Hamid H. M. A., Çelik-Butler Z. (2018). Characterization
and performance
analysis of Li-doped ZnO nanowire as a nano-sensor and nano-energy
harvesting element. Nano Energy.

[12] Wang J., Pan H., Xu X., Jin H., Ma W., Xiong S., Bao Q., Tang Z., Ma Z. (2022). Li-Doped ZnO
Electron Transport Layer
for Improved Performance and Photostability of Organic Solar Cells. ACS Appl. Mater. Interfaces.

[13] Kurudirek S. V., Hertel N. E., Klein B. D. B., Biber M., Summers C. J. (2016). Development
of ZnO Nanorod-Based Scintillators Grown Under a Low-Temperature Hydrothermal
Method for Use in Alpha-Particle and Thermal Neutron Detectors. IEEE Trans. Nucl. Sci..

[14] Kurudirek S. V., Menkara H., Klein B. D. B., Hertel N. E., Summers C. J. (2018). Effect
of annealing temperature on the photoluminescence and scintillation
properties of ZnO nanorods. Nucl. Instrum. Methods
Phys. Res., Sect. A.

[15] Kurudirek S. V., Kurudirek M., Klein B. D. B., Summers C. J., Hertel N. E. (2018). Synthesis
and photoluminescence properties of Ga-doped ZnO nanorods by a low
temperature solution method. Nucl. Instrum.
Methods Phys. Res., Sect. A.

[16] Chen J.-X., Hao S.-T., Sun Z.-X., Zheng P., Tang J., Yang Y.-L., Zhang S.-L., Liu X.-L., Zhao J.-T., Li Q.-L., Zhang Z.-J. (2022). Development
of the ZnO:Ga nanorod
arrays as an alpha particle scintillation screen for the associated
particle neutron generator. Appl. Phys. Lett..

[17] Kurudirek M., Kurudirek S. V., Hertel N. E., Erickson A., Sellin P. J., Mukhopadhyay S., Astam A., Summers C. J. (2023). Vertically Well-Aligned
ZnO Nanoscintillator Arrays with Improved Photoluminescence and Scintillation
Properties. Materials.

[18] Sahani R. M., Pandya A., Dixit A. (2023). ZnO-(6)­LiF/polystyrene composite
scintillator for thermal neutron radiation detection. Rev. Sci. Instrum..

[19] Mann S. E., Schooneveld E. M., Rhodes N. J., Liu D., Sykora G. J. (2023). Position
sensitive ZnO:Zn neutron detector – A high count rate alternative
to ZnS:Ag scintillation detectors. Nucl. Instrum.
Methods Phys. Res., Sect. A.

[20] Sun S., Xiong B., Huang Q., Wan B., Yang D., Wang M., Luo Y., Xia Y., Wei W., Ge X., Chen J. (2024). Research on data processing technology
based on 6LiF/ZnO:Ga
fiber neutron detector array. J. Instrum..

[21] Xiong B., Wan B., Zhao Y., Yang D., Ge X., Sun S. (2024). An optimization
design for fiber-optic neutron detector based on 6LiF/ZnO:Ga and wavelength
shifting fibers. J. Instrum..

[22] Giasari A. S., Maharani Muharam A. P., Syampurwadi A., Dedi, Eddy D. R., Primadona I. (2023). Morphological
effect of one-dimensional ZnO nanostructures on the photocatalytic
activity. J. Phys. Chem. Solids.

[23] Khan W., Khan F., Ajmal H. M. S., Huda N. U., Kim J. H., Kim S. D. (2018). Evolution of Structural and Optical
Properties of ZnO
Nanorods Grown on Vacuum Annealed Seed Crystallites. Nanomaterials.

[24] Lotgering F. K. (1959). Topotactical
reactions with ferrimagnetic oxides having hexagonal crystal structures—I. J. Inorg. Nucl. Chem..

[25] Kung C. Y., Lin C. C., Young S. L., Horng L., Shih Y. T., Kao M. C., Chen H. Z., Lin H. H., Lin J. H., Wang S. J., Li J. M. (2013). Influence of Li
doping on the optical
and magnetic properties of ZnO nanorods synthesized by low temperature
hydrothermal method. Thin Solid Films.

[26] Kerasidou A. P., Mageiras J., Bardakas A., Psycharis V. P., Tsamis C. (2019). Influence of Li-nitrate doping on
the hydrothermally
grown ZnO nanorods. IOSR J. Appl. Phys..

[27] Ko W., Lee S., Myoung N., Hong J. (2016). Solution processed vertically stacked
ZnO sheet-like nanorod p–n homojunctions and their application
as UV photodetectors. J. Mater. Chem. C.

[28] Sharma A., Chakraborty M., Thangavel R., Udayabhanu G. (2018). Hydrothermal
growth of undoped and boron doped ZnO nanorods as a photoelectrode
for solar water splitting applications. J. Sol-Gel
Sci. Technol..

[29] Kim S., Nam G., Park H., Yoon H., Lee S.-H., Kim J. S., Kim J. S., Kim D. Y., Kim S.-O., Leem J.-Y. (2013). Effects
of Doping with Al, Ga, and In on Structural and Optical Properties
of ZnO Nanorods Grown by Hydrothermal Method. Bull. Korean Chem. Soc..

[30] Ruankham P., Sagawa T., Sakaguchi H., Yoshikawa S. (2011). Vertically
aligned ZnO nanorods doped with lithium for polymer solar cells: defect
related photovoltaic properties. J. Mater. Chem..

[31] Luo J., Liu H., Deng W., Zhang R. (2024). The effects of nitrogen ionization
during preparation and oxygen pressure during annealing on the morphology,
structure, and luminescent properties of Mg-doped ZnO thin films. Appl. Phys. A: Mater. Sci. Process..

[32] Burstein E. (1954). Anomalous
Optical Absorption Limit in InSb. Phys. Rev..

[33] Mukherjee S., Pramanik S., Das S., Mandal R., Chakraborty S., Chattopadhyay A., Ghosh T., Pal S., Nath R., Kuiri P. K. (2023). Structural,
optical, and antibacterial properties of
Li-doped ZnO nanoparticles synthesized in water: evidence of incorporation
of interstitial Li. Phys. Scr..

[34] Agrawal J., Dixit T., Palani I. A., Ramachandra
Rao M. S., Singh V. (2018). Fabrication of high responsivity
deep UV photo-detector based on
Na doped ZnO nanocolumns. J. Phys. D: Appl.
Phys..

[35] Tauc, J. Amorphous and Liquid Semiconductors; Springer Science & Business Media, 2012.

[36] He S., Cao S., Liu Y., Chen W., Lyu P., Li W., Bao J., Sun W., Kan C., Jiang M., Liu Y. (2025). Giant Photoluminescence
Enhancement of Ga-Doped ZnO Microwires by X-Ray Irradiation. Adv. Sci..

[37] Chang J., Lin Z., Zhu C., Chi C., Zhang J., Wu J. (2013). Solution-processed
LiF-doped ZnO films for high performance low temperature field effect
transistors and inverted solar cells. ACS Appl.
Mater. Interfaces.

[38] Fonoberov V. A., Alim K. A., Balandin A. A., Xiu F., Liu J. (2006). Photoluminescence
investigation of the carrier recombination processes in ZnO quantum
dots and nanocrystals. Phys. Rev. B.

[39] Yang Y. H., Chen X. Y., Feng Y., Yang G. W. (2007). Physical mechanism
of blue-shift of UV luminescence of a single pencil-like ZnO nanowire. Nano Lett..

[40] Li Q., Hao S., An R., Wang M., Sun Z., Wu Q., Gu M., Zhao J., Liu X., Zhang Z. (2019). Ultraviolet-light emission
enhancement and morphology stability for ZnO:Ga nanorod array treated
by hydrogen plasma. Appl. Surf. Sci..

[41] Parashar S. K. S., Murty B. S., Repp S., Weber S., Erdem E. (2012). Investigation
of intrinsic defects in core-shell structured ZnO nanocrystals. J. Appl. Phys..

[42] Erdem E. (2014). Microwave
power, temperature, atmospheric and light dependence of intrinsic
defects in ZnO nanoparticles: A study of electron paramagnetic resonance
(EPR) spectroscopy. J. Alloys Compd..

[43] Hong M., Meng J., Yu H., Du J., Ou Y., Liao Q., Kang Z., Zhang Z., Zhang Y. (2021). Ultra-stable
ZnO nanobelts in electrochemical environments. Mater. Chem. Front..

[44] Parks D. A., Tittmann B. R. (2014). Radiation tolerance of piezoelectric bulk single-crystal
aluminum nitride. IEEE Trans. Ultrason. Ferroelectr.
Freq. Control.

[45] Koike K., Aoki T., Fujimoto R., Sasa S., Yano M., Gonda S. i., Ishigami R., Kume K. (2012). Radiation hardness
of single-crystalline zinc oxide films. Phys.
Status Solidi C.

[46] Stevie F. A., Donley C. L. (2020). Introduction to x-ray photoelectron spectroscopy. J. Vac. Sci. Technol..

[47] Huang C.-H., Chu Y.-L., Ji L.-W., Tang I. T., Chu T.-T., Chiou B.-J. (2022). Fabrication and
characterization of homostructured
photodiodes with Li-doped ZnO nanorods. Microsyst.
Technol..

[48] Fabbri F., Villani M., Catellani A., Calzolari A., Cicero G., Calestani D., Calestani G., Zappettini A., Dierre B., Sekiguchi T., Salviati G. (2014). Zn vacancy induced green luminescence on non-polar
surfaces in ZnO nanostructures. Sci. Rep..

[49] Foley M., Ton-That C., Phillips M. R. (2008). Cathodoluminescence inhomogeneity
in ZnO nanorods. Appl. Phys. Lett..

[50] Chen L., Ruan J., Xu M., He S., Hu J., Zhang Z., Liu J., Ouyang X. (2019). Comparative study on
fluorescence decay time of doped ZnO crystals under α and β
excitation. Nucl. Instrum. Methods Phys. Res.,
Sect. A.

[51] Iyer V., Roccapriore K., Ng J., Srijanto B., Lingerfelt D., Lawrie B. (2023). Photon bunching in
cathodoluminescence induced by indirect
electron excitation. Nanoscale.

[52] Feldman M. A., Dumitrescu E. F., Bridges D., Chisholm M. F., Davidson R. B., Evans P. G., Hachtel J. A., Hu A. M., Pooser R. C., Haglund R. F., Lawrie B. J. (2018). Colossal photon bunching in quasiparticle-mediated
nanodiamond cathodoluminescence. Phys. Rev.
B.

[53] Koida T., Chichibu S. F., Uedono A., Tsukazaki A., Kawasaki M., Sota T., Segawa Y., Koinuma H. (2003). Correlation
between the photoluminescence lifetime and defect density in bulk
and epitaxial ZnO. Appl. Phys. Lett..

[54] Chen X., Zhang Z.-C., Zhang K., Guan X.-Y., Weng X.-F., Han H.-T. (2020). Study on the Time
Response of a Barium Fluoride Scintillation
Detector for Fast Pulse Radiation Detection. IEEE Trans. Nucl. Sci..

[55] Li Y., Shao W., Chen L., Wang J., Nie J., Zhang H., Zhang S., Gao R., Ouyang X., Ouyang X., Xu Q. (2021). Lead-halide Cs4PbBr6 single crystals
for high-sensitivity radiation detection. NPG
Asia Mater..

[56] Lin R., Guo Q., Zhu Q., Zhu Y., Zheng W., Huang F. (2019). All-Inorganic
CsCu­(2) I(3) Single Crystal with High-PLQY (approximately 15.7%) Intrinsic
White-Light Emission via Strongly Localized 1D Excitonic Recombination. Adv. Mater..

[57] Liu J., Liu F., Ouyang X., Liu B., Chen L., Ruan J., Zhang Z., Liu J. (2013). The luminescence characteristics
of CsI­(Na) crystal under α and X/γ excitation. J. Appl. Phys..

[58] Min S., Ko K.-H., Seo B., Roh C., Hong S. (2022). Integration
of Decay Time Analysis and Radiation Measurement for Quantum-Dot-Based
Scintillator’s Characterization. Processes.

[59] Shibata M., Sekiya H., Ichimura K. (2022). Precise measurement of the scintillation
decay constant of the ZnWO4 crystal. Prog. Theor.
Exp. Phys..

[60] Wen X., Enqvist A. (2017). Measuring the scintillation
decay time for different
energy deposited by γ-rays and neutrons in a Cs2LiYCl6:Ce3+
detector. Nucl. Instrum. Methods Phys. Res.,
Sect. A.

[61] Yamamoto S., Tomita H. (2021). Comparison of light
outputs, decay times, and imaging
performance of a ZnS­(Ag) scintillator for alpha particles, beta particles,
and gamma photons. Appl. Radiat. Isot..

[62] Bessiere A., Dorenbos P., van Eijk C. W. E., Kramer K. W., Gudel H. U. (2004). New thermal
neutron scintillators: Cs/sub 2/LiYCl/sub 6/:Ce/sup 3+/and Cs/sub
2/LiYBr/sub 6/:Ce/sup 3+. IEEE Trans. Nucl.
Sci..

[63] Xie A., Hettiarachchi C., Maddalena F., Witkowski M. E., Makowski M., Drozdowski W., Arramel A., Wee A. T. S., Springham S. V., Vuong P. Q., Kim H. J., Dujardin C., Coquet P., Birowosuto M. D., Dang C. (2020). Lithium-doped two-dimensional
perovskite scintillator for wide-range radiation detection. Commun. Mater..

[64] Lin R., Zhu Y., Chen L., Zheng W., Xu M., Ruan J., Li R., Li T., Lin Z., Cheng L., Ding Y., Huang F., Ouyang X. (2022). Ultrafast (600 ps) α-ray scintillators. PhotoniX.

[65] Chen L., He S.-Y., Zhou L.-D., Huang F., Hu J., Ruan J.-L., Xu M.-X., Zhang Z.-B., Liu J.-L., Ouyang X.-P., Liu B. (2019). The dependence
of fluorescent decay
time of ZnO:Ga crystal on instantaneous non-equilibrium carriers induced
by charged particles. J. Lumin..

[66] Shinohara K., Agulto V. C., Empizo M. J. F., Yamanoi K., Shimizu T., Nakajima M., Yoshimura M., Salvador A. A., Fukuda T., Sarukura N. (2024). Gamma-ray irradiation
and the alterations in photoluminescence
emissions of undoped and indium-doped ZnO single crystals. J. Cryst. Growth.

[67] Angub M. C. M., Vergara C. J. T., Husay H. A. F., Salvador A. A., Empizo M. J. F., Kawano K., Minami Y., Shimizu T., Sarukura N., Somintac A. S. (2018). Hydrothermal growth
of vertically
aligned ZnO nanorods as potential scintillator materials for radiation
detectors. J. Lumin..

[68] Fiedler S., Ton-That C., Phillips M. R. (2023). Defect-free ZnO
nanorods with high
angular distribution for enhanced excitonic emission. J. Mater. Res..

[69] Sahani R. M., Pandya A., Dixit A. (2021). Zinc oxide/polystyrene
composite
based scintillator for alpha particle monitoring. Mater. Sci. Semicond. Process..

[70] Ziegler J. F., Ziegler M. D., Biersack J. P. (2010). SRIM –
The stopping and range
of ions in matter. Nucl. Instrum. Methods Phys.
Res., Sect. B.

