# Supplementary material for: Vertically Self-Oriented, Ultrafast 1D ZnO:Li Nanorods as Scintillators for Thermal Neutron Detection
Source: ACS Appl Nano Mater. 2025 Oct 17;8(43):20697–712. doi: 10.1021/acsanm.5c03082 (PMC12584104; doi:10.1021/acsanm.5c03082)
Supplement: Supplementary file 1 [file an5c03082_si_001.pdf]

Supporting Information

Vertically Self-Oriented, Ultrafast 1D ZnO:Li Nanorods as Scintillators for Thermal Neutron Detection

Murat Kurudirek[1,2,3], Sinem V. Kurudirek[1], Anna Erickson[1], Paul J. Sellin[2], Mackenzie Duce[1], Johan Gouws[1], Benjamin J Lawrie[4,5], Charles L. Melcher[6,8,9], Nolan Hertel[1]*

[1] Nuclear and Radiological Engineering Program, G. W. Woodruff School of Mechanical Engineering, Georgia Institute of Technology, Atlanta, GA 30332, USA
[2] School of Mathematics and Physics, University of Surrey, Guildford GU2 7XH, UK
[3]Department of Electricity and Energy, Technical Sciences Vocational College, Ataturk University, Erzurum 25240, Turkey
[4]Center for Nanophase Materials Sciences, Oak Ridge National Laboratory, Oak Ridge, TN 37831, USA
[5]Materials Science and Technology Division, Oak Ridge National Laboratory, Oak Ridge, TN 37831, USA
[6]Scintillation Materials Research Center, University of Tennessee, Knoxville, TN, 37996, USA
[8]Department of Nuclear Engineering, University of Tennessee, Knoxville, TN, 37996, USA
[9]Department of Materials Science and Engineering, University of Tennessee, Knoxville, TN, 37996, USA

Email: mkurudirek@atauni.edu.tr

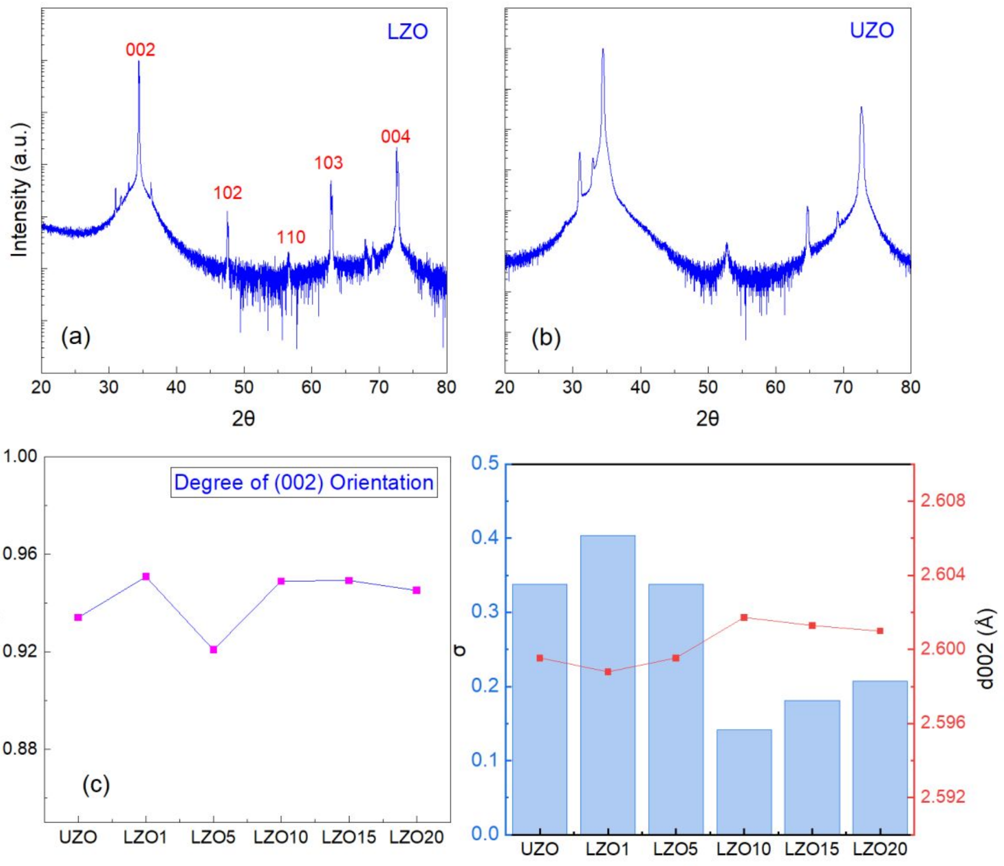

Figure S1 The enlarged XRD patterns of a) LZO (10%), b) UZO, c) the degree of c-axis alignment of ZnO NRs. d) d-spacing and stress in ZnO NRs

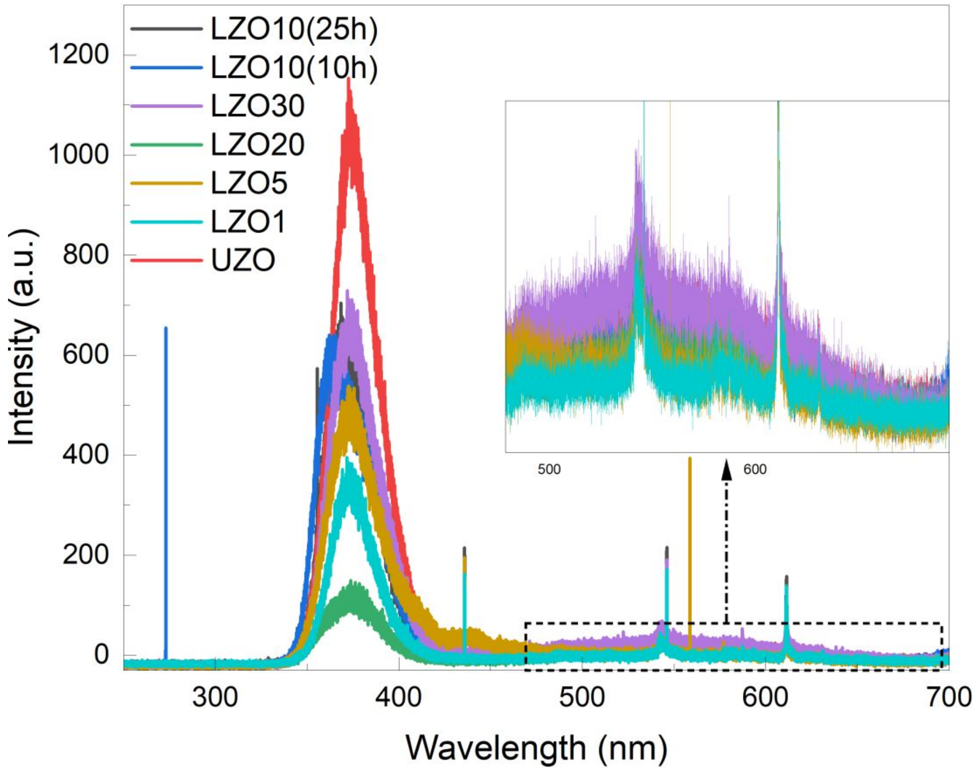

Figure S2. PL intensity of the ZnO NRs before annealing in a forming gas atmosphere (10% H2).

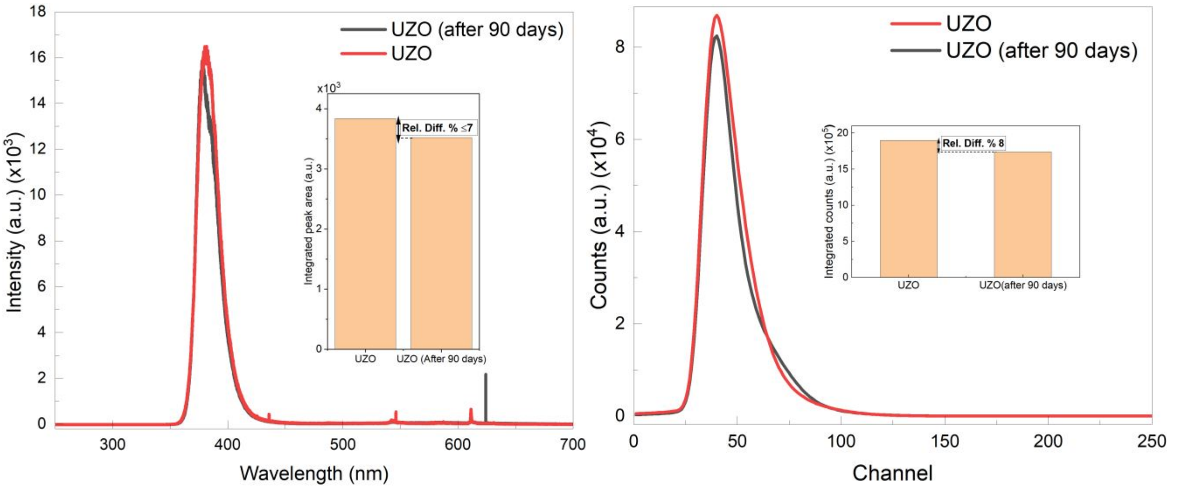

Figure S3. a) PL intensity, b) alpha response of the UZO NRs after an ageing period of 90 days.

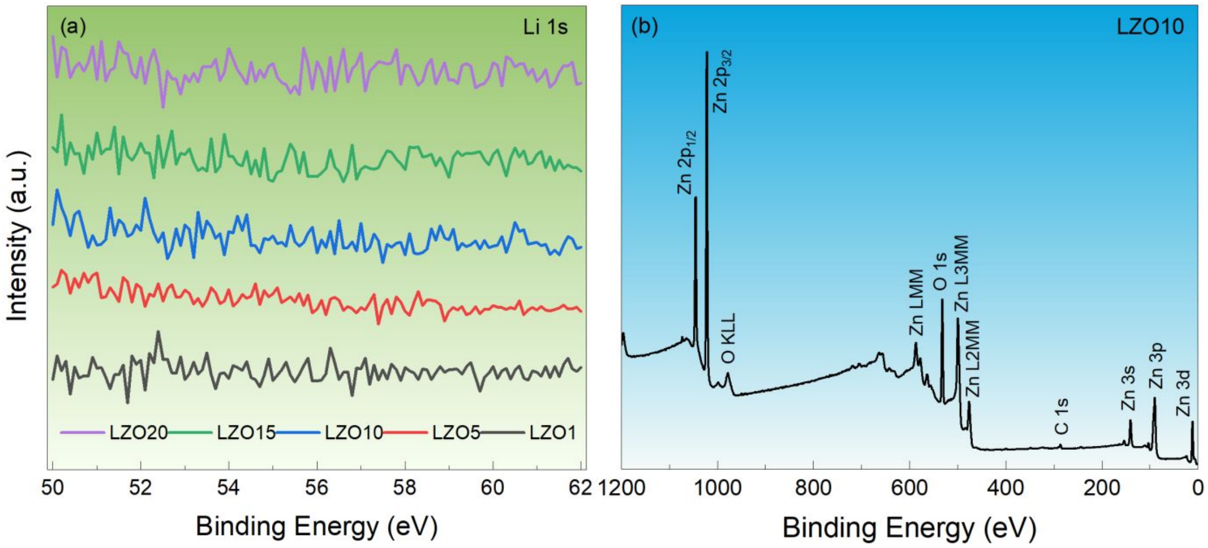

Figure S4. a) Li 1s XPS spectrum of the 1%, 5%, 10%, and 20% LZO NRs. b) The XPS survey spectra of LZO (10%) NRs.

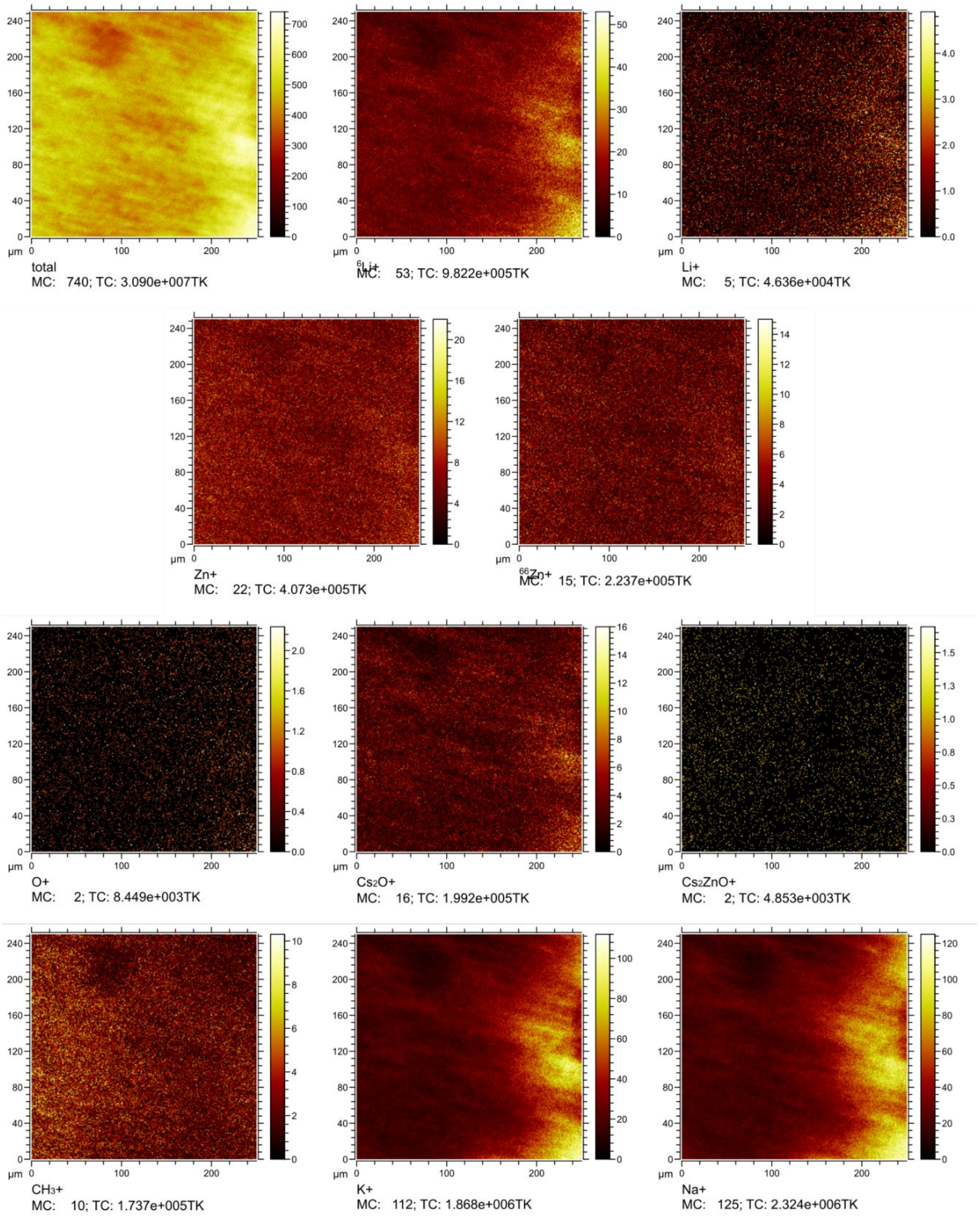

Figure S5. The ToF-SIMS results for the LZO (10% 6Li) NRs.

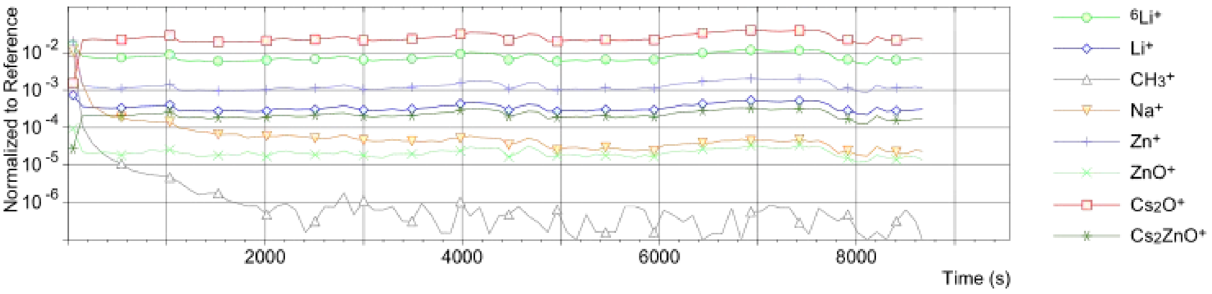

Figure S6. The ToF-SIMS depth profile for the LZO (10% 6Li) NRs. The Cs compounds arise from sputtering reactions on the sample surface. Almost all the sample species remain constant over a long cumulative sputtering period. The surface contaminants (CH3+ and Na+) decrease rapidly as probing deeper into the sample.

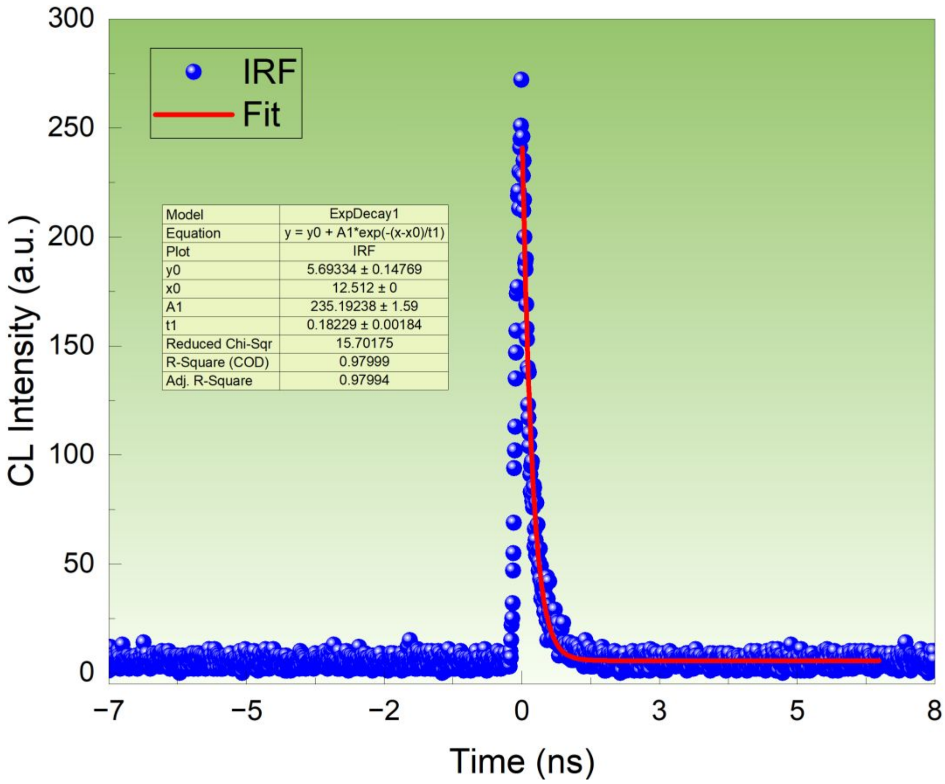

Figure S7. Temporal response of a transition radiation from a gold thin film which is used as the instrument response function (IRF ≈ 180 ps) in CL photon correlation measurements.

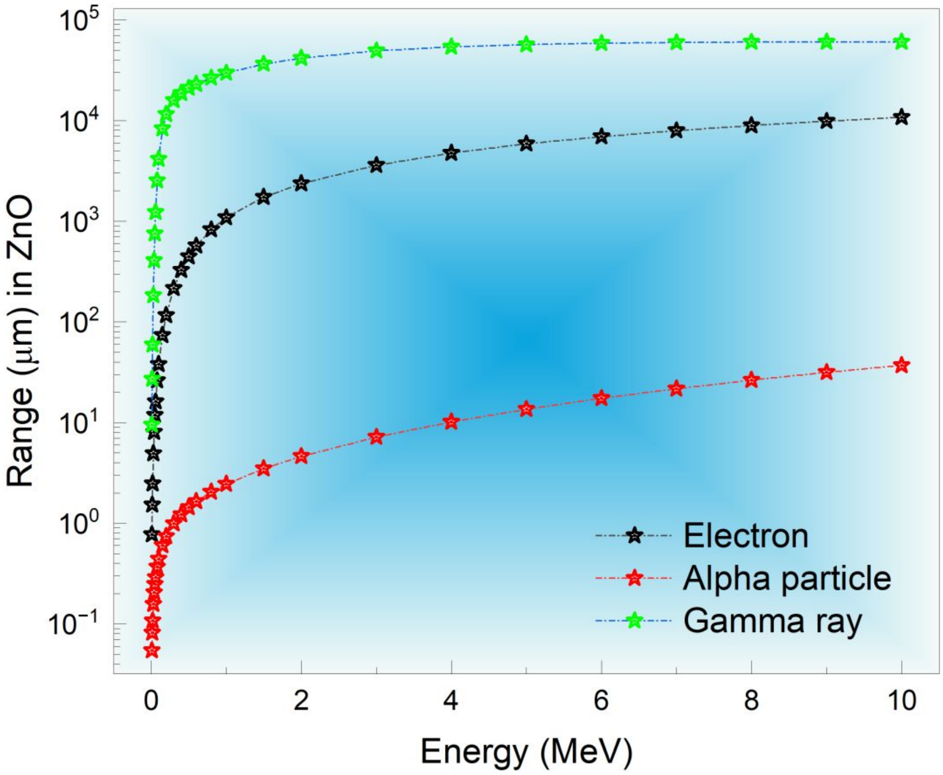

Figure S8. Range (μm) of gammas, electrons, and alpha particles in ZnO.
